# White matter analysis of the extremely preterm born adult brain

**DOI:** 10.1016/j.neuroimage.2021.118112

**Published:** 2021-08-15

**Authors:** Hassna Irzan, Erika Molteni, Michael Hütel, Sebastien Ourselin, Neil Marlow, Andrew Melbourne

**Affiliations:** a Dept. Medical Physics and Biomedical Engineering, University College London, United Kingdom; b School of Biomedical Engineering and Imaging Sciences, Kings College London, United Kingdom; c Institute for Women’s Health, University College London, United Kingdom

**Keywords:** Diffusion MRI, Graph theory, Prematurity, Brain development, White matter, Structural connectome, Brain microstructure, Long-term prematurity outcome

## Abstract

The preterm brain has been analysed after birth by a large body of neuroimaging studies; however, few studies have focused on white matter alterations in preterm subjects beyond infancy, especially in individuals born at extremely low gestation age - before 28 completed weeks. Neuroimaging data of extremely preterm young adults are now available to investigate the long-term structural alterations of disrupted neurodevelopment. We examined white matter hierarchical organisation and microstructure in extremely preterm young adults. Specifically, we first identified the putative hubs and peripheral regions in 85 extremely preterm young adults and compared them with 53 socio-economically matched and full-term born peers. Moreover, we analysed Fractional Anisotropy (FA), Mean Diffusivity (MD), Neurite Density Index (NDI), and Orientation Dispersion Index (ODI) of white matter in hubs, peripheral regions, and over the whole brain. Our results suggest that the hierarchical organisation of the extremely preterm adult brain remains intact. However, there is evidence of significant alteration of white matter connectivity at both the macro- and microstructural level, with overall diminished connectivity, reduced FA and NDI, increased MD, and comparable ODI; suggesting that, although the spatial configuration of WM fibres is comparable, there are less WM fibres per voxel. These alterations are found throughout the brain and are more prevalent along the pathways between deep grey matter regions, frontal regions and cerebellum. This work provides evidence that white matter abnormalities associated with the premature exposure to the extrauterine environment not only are present at term equivalent age but persist into early adulthood.

## Highlights

• We investigated the long-term effect of extreme prematurity on structural and microstructural characteristics of the brain network.
• The hierarchical organisation of the extremely preterm adult brain is intact; however, the extremely preterm brain has significantly reduced structural connectivity and neurite density compared to the term brain.
• The most significant alterations are observed in the somatosensory and motor cortical areas, and in the deep nuclei.

## 1 Introduction

Preterm babies are born at fewer than 37 completed weeks of gestation. In 2010, the World Health Organization estimates that more than one in ten babies are born preterm worldwide, and this number is rising Organization (2012). In developed countries, better socioeconomic factors and improved neonatal care have strengthened the ability to treat babies born at immature gestational ages; increased survival rates, however, have not paired with equal decrease in neonatal morbidity Costeloe et al. (2012); Moore et al. (2012); Organization (2012) and reduction of cases with cognitive impairment. In fact, it is well established that decline in cognitive performance is associated with preterm birth Ball et al. (2015); Hedderich et al. (2020); Marlow et al. (2005); Moore et al. (2012); O’Reilly et al. (2020); Sølsnes et al. (2016). Survivors suffer complications in multiple organs, of which brain and lungs are the most vulnerable Saigal and Doyle (2008). Effects are not limited only to infancy but extend in the long term Saigal and Doyle (2008). This is of great concern since the absolute number of subjects with a broad spectrum of neurologic and cognitive disability is increasing, with associated costs for society, health care systems, and education Frey and Klebanoff (2016). Evaluating the extent of any impairment, and identifying which brain areas or neurological networks are most compromised enables prediction of the long-term effect of prematurity on the brain. This is crucial to inform personalised treatments and targeted therapies at an early stage.

Evidence suggests that the preterm brain manifests anatomical Ball, Aljabar, Zebari, Tusor, Arichi, Merchant, Robinson, Ogundipe, Rueckert, Edwards, Counsell, 2014, Ball, Boardman, Rueckert, Aljabar, Arichi, Merchant, Gousias, Edwards, Counsell, 2012; Hedderich et al. (2020), microstructural Ball et al. (2013b) and functional impairments Moore et al. (2012); O’Reilly et al. (2020) from early years. Although diffuse White Matter (WM) abnormalities are the most common hallmark of the prematurity Ball et al. (2012), early exposure to the extrauterine environment is also associated with long-term alterations of the brain’s cortical complexity Hedderich et al. (2020), smaller subcortical and cerebral WM volumes, and larger ventricles Sølsnes et al. (2016). Findings from Magnetic Resonance Imaging (MRI) suggest that core WM connectivity may be formed before the time of normal birth, establishing a backbone structure for the formation of additional and later connectivity Ball et al. (2014). Diffusion MRI studies on preterm infants reveal that this process is influenced by premature birth, resulting in disrupted cortical-subcortical WM connectivity Ball et al. (2014) and microstructure Ball et al. (2013b). In addition, WM connectivity in highly connected brain areas such as hubs and rich club regions Zhao et al. (2019) and connections to and from deep grey matter areas Batalle et al. (2017) exhibit most prominent maturation with increasing gestational age. The preterm brain, having to cope with a scarcity of WM resources, might therefore favour the establishment and the maturation of core connectivity at the expense of peripheral connectivity Batalle et al. (2017); Karolis et al. (2016). Sudden interruption of typical brain maturation after preterm birth alters the development of WM structure Ball, Aljabar, Zebari, Tusor, Arichi, Merchant, Robinson, Ogundipe, Rueckert, Edwards, Counsell, 2014, Ball, Boardman, Rueckert, Aljabar, Arichi, Merchant, Gousias, Edwards, Counsell, 2012 leading to altered network architecture, reduced network capacity Ball et al. (2014), decreased global efficiency, decreased network integration, and increased network segregation de Almeida et al. (2021). In addition, earlier gestational age is associated with lower weighted nodal strength, characteristic path length, clustering coefficient Brown et al. (2014), global, and local efficiency Batalle et al. (2017). The preterm brain is characterised by altered cortical microstructure inferred by changes in Fractional Anisotropy (FA) and Mean Diffusivity (MD) at term-equivalent age Ball et al. (2013b), and reduced FA in major WM matter tracts in childhood and early adulthood Batalle et al. (2017); Eikenes et al. (2011); Fischi-Gómez et al. (2015); Young et al. (2019). Orientation Dispersion Index (ODI) and Neurite Density Index (NDI) parameters are significantly altered in preterm born neonates Batalle et al. (2017), children Young et al. (2019), and adolescents Eaton-Rosen et al. (2016).

The microstructure and the WM tract architecture is characterised by Diffusion Tensor Imaging (DTI). Models such as spherical deconvolution Jeurissen et al. (2014); Tournier et al. (2007) and Neurite Orientation Dispersion and Density Imaging (NODDI) Zhang et al. (2012), which have higher specificity and biological plausibility, are available to overcome some of the inherent limitations of the diffusion tensor Jones and Cercignani (2010). NODDI proposes three compartments to model the intra-cellular, extra-cellular, and cerebrospinal fluid environments. To characterise WM, two parameters are of interest: ODI and NDI. As a result, NODDI offers the prospect of improved specificity when measuring changes in tissue microstructure and network architecture related to prematurity.

The majority of studies investigate the preterm brain in the early stages after birth Ball, Aljabar, Zebari, Tusor, Arichi, Merchant, Robinson, Ogundipe, Rueckert, Edwards, Counsell, 2014, Ball, Boardman, Rueckert, Aljabar, Arichi, Merchant, Gousias, Edwards, Counsell, 2012, Ball, Srinivasan, Aljabar, Counsell, Durighel, Hajnal, Rutherford, Edwards, 2013 and in childhood Young et al. (2019), while less is known about subjects beyond childhood Eikenes et al. (2011); Fischi-Gómez et al. (2015), especially extremely preterm born individuals Eaton-Rosen et al. (2016); Irzan, Hütel, Semedo, O’Reilly, Sahota, Ourselin, Marlow, Melbourne, 2020, Irzan, O’Reilly, Ourselin, Marlow, Melbourne, 2020. Therefore, as the long-term effect of preterm birth on the brain is incompletely explored and not fully understood, it remains unclear how the pathophysiology of early WM impairment impacts brain architecture in adulthood and how brain abnormalities of early life evolve into adulthood. In this study, we address the lack of long-term studies and investigate the impact of extremely preterm birth on the development of whole-brain WM. The analysis is carried out using a graph-based description, which enables the application of graph theory measures. With such description, the brain is represented as a graph with nodes denoting the brain regions and edges the WM connectivity. Graph theory measures describe anatomical properties of the brain and have been observed to quantify differences between patient and control groups Bullmore and Sporns (2009).

Hubs are highly connected brain regions with a crucial role in the transfer of information in the network and are therefore closely linked to the concept of centrality Rubinov and Sporns (2010). Brain regions are usually defined as either hub or peripheral regions depending on the specific centrality measure used. Since each centrality measure reflects a region’s topological characteristic, different types of hubs or peripheral regions can be specified. For example, defining hubs based on high nodal strength would identify regions that connect to many brain regions, defining hubs following high nodal efficiency would identify regions that mediate network integration and delivery of information, and defining hubs according to high betweenness centrality would identify regions that lie on many shortest path. To identify hubs with several topological characteristics, we use a consensus-based approach van den Heuvel et al. (2010) by combining rankings across centrality measures. Since hubs are essential to maintain network wide-information flow, a potential dysfunction or loss after preterm birth might have disproportional effects on the integrity and functionality of the remainder of the network. Although the presence of adult-like brain network architecture is observed in infants before term birth Van Den Heuvel et al. (2015), including the existence of the small-world modular organisation and the presence of hubs Ball et al. (2014), the developing preterm brain may be required to reorganise its WM connectivity due to its anatomical limitation. Hence, the identification of hubs and peripheral regions would inform about the long-term effects of extreme prematurity on the brain’s hierarchical architecture. We investigate the microstructural parameters (FA, MD, ODI, NDI) along these tracts and test the hypothesis that extreme prematurity is associated with altered microstructural outcome. Finally, we investigate the extent to which the alteration of the microstructural properties might be linked to gestational age and inspect which sub-networks of the brain are mainly affected.

## 2 Methods

**Fig. 1:**
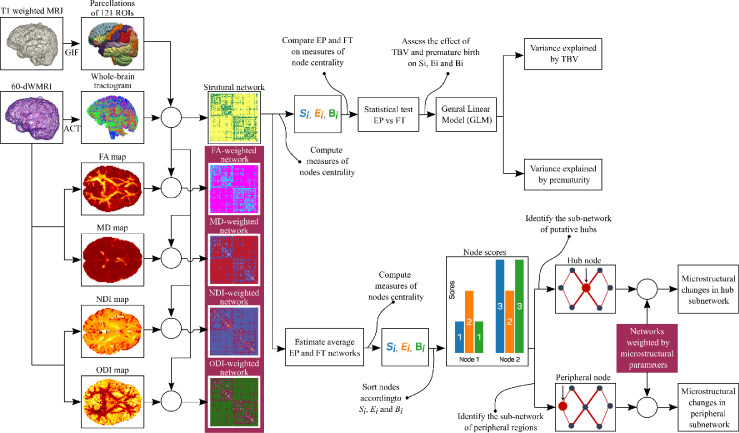
Outline of the main steps of the methodology to estimate and analyse the structural networks and networks weighted by the microstructural parameters FA, MD, NDI, and ODI. We use Anatomically Constrained Tractography (ACT) Smith et al. (2012) to evaluate the white matter streamlines. We evaluate the structural network by quantifying the white matter connectivity between brain areas obtained from Geodesic Information Flow (GIF) parcellations Cardoso et al. (2015) . We measure maps of the microstructural parameters and estimated FA, MD, NDI, and ODI -weighted networks. We compare Extremely Preterm (EP) and Full-Term (FT) born subjects on measures of node centrality ($S_{i},E_{i},B_{i}$) and investigate how these measures are influenced by Total Brain Volume (TBV) and being born extremely preterm. Moreover, by combining rankings of $S_{i},E_{i},\text{and}\;B_{i}$, we estimate hub and peripheral brain regions on the average EP and FT network. In addition, we evaluate the microstructural parameters along the hub and peripheral sub-network, and over all the brain.

We describe the participants and data acquisition in Section 2.1, the steps to complete data preprocessing in Section 2.2, the steps to perform tractography in Section 2.3, and microstructural features estimation in Section 2.4. Section 2.5 outlines how we calculate the structural network, and the microstructural networks weighted by FA, MD, NDI, and ODI. In Section 2.6, we introduce the node centrality measures and compare them between a group of Extremely Preterm born subjects (EP) and a group of Full-Term born subjects (FT) in Section 2.7. We use nodes centrality measures to perform identification of hub regions in Section 2.8 and peripheral regions in Section 2.9. Moreover, we investigate the microstructural features along these sub-networks in Section 2.10. Fig. 1 outlines the main steps of the methodology.

### 2.1 Participants and data acquisition

One hundred thirty-eight participants are recruited from the EPICure study of infants born extremely preterm at 26 completed weeks of gestation or less between March and December 1995 in the United Kingdom and the Republic of Ireland. The cohort includes a group of 53 FT who are socio-economically matched with 85 EP O’Reilly et al. (2020). Table 1 reports the details of the cohort.

**Table 1:** Characteristics of the participants with the total number of Extremely Preterm (EP) and Full-Term (FT) born subjects, the proportion of females and males in the cohort, and the number of subjects with respect to their gestational age at birth in completed Weeks (W).

Participants characteristics	EP	FT
Number of subjects N	85	53
Sex (female/male)	49/36	32/21
Completed Weeks of gestation		
23:= 22–23 W (female/male)	7/2	−/−
24:= 24 W (female/male)	14/8	−/−
25:= 25 W (female/male)	28/26	−/−
40:= from 37 W (female/male)	−/−	32/21

T1-weighted MRI and diffusion weighted MRI (dWMRI) acquisitions are performed on a 3T Philips Achieva system at subjects’ 19^th^ birthday. MPRAGE T1-weighted MRI images are acquired at Repetition Time (TR) of 6.93 ms, Echo Time (TE) of 3.14 ms with 1 mm isotropic resolution. Volumes of dWMRI are acquired at (2.5×2.5×3) mm resolution across b-values of (0,300,700,2000) s/mm${}^{2}$, and n: 4,8,16,32 directions. The acquisitions are performed at TE: 70 ms, TR: 3500 ms, field of view: (240×240×150) mm, flip angle: $90^{\circ }$, and SENSE factor of one.

Data from three EP are rejected from the analysis because they have disconnected structural networks (i.e. one or more regions are not connected to other brain regions). Note that these subjects do not show an evident WM injury. All participants gave informed consent before taking part in the experiment. Ethical approval was granted by the South Central - Hampshire A Research Ethics Committee (reference 13/ SC/0514).

### 2.2 Data pre-processing

T1-weighted images are bias-corrected using the N4ITK algorithm Tustison et al. (2010). Tissue parcellations of the bias-corrected T1-weighted volumes are obtained using the Geodesic Information Flow (GIF) version 2.0 Cardoso et al. (2015). GIF uses a multi-atlas label propagation and fusion strategy to propagate voxel-wise annotations into each subject’s native space, producing 144 labels. Before inclusion in the study, all the scans and parcellations are visually inspected for assessing the quality of the data. All parcellations are judged acceptable, and no data is rejected at this stage.

The preprocessing of the dWMRI volumes is completed using *MRtrix3* package Tournier et al. (2019). The volumes of dWMRI are first corrected for thermal noise by removing data redundancy based on eigenspectrum of random covariance matrices approach Veraart et al. (2016). Gibbs-ringing artefacts are addressed by sampling the ringing pattern at the zero-crossings of the oscillating sinc-function Kellner et al. (2016). Furthermore, dWMRI volumes are corrected for eddy current-induced distortion and subject movements artefacts by comparing the predicted dWMRI volumes to the observed data; the resulting difference updates the estimate of the eddy-current-induced field and the position of the subject Andersson and Sotiropoulos (2016). In addition, we compare head motion between EP and FT. Therefore, we first estimate head displacement and rotation for each subject by calculating the maximum translation and rotation in each scan; we then compare these between EP and FT. The statistical test for difference in the mean shows that there is no evidence of a significant difference in displacement (p=.49) or rotation (p=.11) between the two groups.

### 2.3 Tractography

The response function of each tissue type (cerebrospinal fluid, WM, and GM) is estimated using a Multi-Shell Multi-Tissue (MSMT) approach Jeurissen et al. (2014). The orientation distribution of the WM fibres (FOD) is then evaluated in each voxel through Constrained Spherical Deconvolution (CSD) Tournier et al. (2007). The intensity of the FODs is normalised for inter-subject comparisons. Whole-brain streamlines probabilistic tractography is performed using dynamic seeding with backtrack Smith et al. (2015). Within the dynamic seeding strategy, the difference between the reconstructed streamlines density and the FOD amplitudes is employed to update the probability of seeding from each FOD lobe dynamically. To ensure that the tracts terminate in GM, the seed location is checked by the Anatomically Constrained Tractography (ACT) framework Smith et al. (2012). The estimated tracks consist of ten million streamlines per subject. To best approximate the apparent fibre density, the tracks are filtered with Spherical-deconvolution Informed Filtering of Tractograms (SIFT2) procedure Smith et al. (2015). The SIFT2 algorithm ascribes weights to each streamline to achieve proportional correspondence between FOD and reconstructed streamlines. Computation of the whole-brain tractogram and brain network estimation are performed using *MRtrix3* package Tournier et al. (2019).

### 2.4 Estimation of microstructural features

Diffusion data are fitted to the DTI Basser et al. (1994) and NODDI Zhang et al. (2012) models. From DTI, the voxel-wise parameters FA and MD are estimated. FA describes the normalised variance of the eigenvalues of the diffusion tensor about their mean; MD is the mean of the eigenvalues of the diffusion tensor. While FA is a measure related to aspects of fibre geometry and integrity, including density, myelination, dispersion, and crossings; MD is the average diffusivity over all directions and it relates to many of the same factors as FA. From NODDI model, NDI and ODI are considered: NDI measures the proportion of the space inside the neurites membrane, while ODI refers to the pattern of neurite orientations Zhang et al. (2012).

### 2.5 Brain networks reconstruction

A total of 121 Regions Of Interest (ROIs) are defined on the basis of GIF Cardoso et al. (2015) parcellation and are located within the neocortex, subcortical structures, cerebellum, pons, and brainstem. Structural white matter pathways are defined between these regions. The structural network is defined based on WM connectivity only. Mathematically, this is specified by a graph $G_{n}=\left(V,L\right)$ with V the set of individual ROIs and L the set of edges (or links) connecting the individual ROIs. Each edge (i,j) connects two brain regions i and j with strength of connectivity $w_{ij}$, which is defined by the weighted (SIFT2 Smith et al. (2015)) contribution of each streamline connecting i and j. For each subject and each microstructural map, a graph $G_{micr}=\left(V,E\right)$ is defined with V the set of individual ROIs and E the set of edges connecting the individual ROIs. Each edge (i,j) connecting a pair of nodes i and j is defined as the mean sampled value of the specific microstructural parameter along each streamline connecting i and j. It is worth noticing that the graphs $G_{n}$ and $G_{micr}$ are isomorphic since they contain the same nodes connected in the same way. Defining the graphs in such a manner guarantees a one-to-one correspondence between the entries of the adjacency matrices associated with each graph for each subject.

### 2.6 Measures of node centrality

The topology of each structural network is investigated. The *weighted connectivity strength* $S_{i}$ of the node i is defined as the sum of edge weights for edges that connect node *i* to all other nodes *j*. Formally:

(1) $$S_{i}=\sum_{i\neq j}w_{ij}$$

The total connectivity strength S is the sum of $S_{i}$ for all the nodes i∈V.

The *weighted nodal efficiency* $E_{i}$ describes how well a brain region is integrated within the whole brain *via* its shortest path $l_{ij}$. The *weighted nodal efficiency* $E_{i}$ of node i is defined as the normalised sum of the reciprocal of the shortest path $l_{ij}$ between node i and all other nodes j∈V:

(2) $$E_{i}=\frac{1}{|V|-1}\sum_{i}\frac{1}{l_{ij}}$$

The *weighted global efficiency* $E_{glob}$ is the normalised sum of the reciprocal of all shortest paths in the network (Latora, Marchiori, 2001),

(3) $$E_{glob}=\frac{1}{\left|V|(|V|-1\right)}\sum_{i\neq j}\frac{1}{l_{ij}}$$

While $E_{glob}$ quantifies how well information flows in a parallel system *via* shortest path over the entire network, $E_{i}$ localises such effect on specific brain regions (Latora, Marchiori, 2001).

The *weighted betweenness centrality* $B_{i}$ is defined as the sum of the ratio of shortest path σ(h,j|i) between any pair of nodes *j* and *h* that passes through node *i* and the overall shortest path σ(h,j) between nodes j and h (Freeman, 1977). This is normalised by the total number of nodes |V| as:

(4) $$B_{i}=\frac{1}{\left(|V|-1)(|V|-2\right)}\sum_{h\neq i,h\neq j,j\neq i}\frac{\sigma (h,j|i)}{\sigma (h,j)}$$

Given the assumption that the information travels through the brain along the shortest path, the brain region with high $B_{i}$ represents a pivotal element in the brain as that region lies on many shortest path and hence mediates a high amount of information traffic.

For simplicity, we omit the term ”weighted” from the graph measures. For each brain network graph $G_{n}$, we compute $S_{i},S,E_{i},E_{glob},\;\text{and,}\;B_{i}$ as defined above.

### 2.7 Statistical analysis

Changes in the node-specific measures of node centrality between FT and EP are examined. Each node is analysed by comparing node-specific $S_{i},E_{i},B_{i}$ distributions between FT and EP using student’s *t*-test. The conducted inferences are corrected for multiple comparisons (Bonferroni correction. We use Cohen’s ds to analyse the effect size for comparing two populations means.

As a previous analysis Irzan et al. (2020d) on this dataset detected brain volume differences between FT and EP, to ensure a meaningful comparison between the two groups and assess the effect of brain volume as well as extreme preterm birth on graph theory measures, we include the Total Brain Volume (TBV) and preterm birth (categorical variable of group membership) as nuisance regressors in a General Linear Model (GLM). The sum of the whole-brain GM and WM volumes, including the cerebellum and the brainstem, represents TBV. GLM is fitted to each graph theory metric using TBV and group membership as covariates.

### 2.8 Consensus identification of hubs

**Fig. 2:**
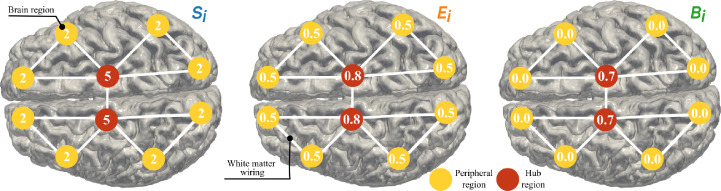
Node-specific values of *connectivity strength*$S_{i}$, *nodal efficiency*$E_{i}$, and *betweenness centrality*$B_{i}$ for a toy network where WM connectivity $w_{ij}$ between two brain regions is one. Under our definition of hub and peripheral regions, hub and peripheral regions are displayed in red and yellow respectively.

Hubs display high level of *connectivity strength* $S_{i}$ van den Heuvel and Sporns (2013), high *betweenness centrality* $B_{i}$ van den Heuvel et al. (2010), and high *nodal efficiency* $E_{i}$ van den Heuvel and Sporns (2013). We derive the average structural network for EP and FT, as their structural network arithmetic average, and compute $S_{i}$, $B_{i}$, and $E_{i}$ of the average EP and FT networks. For each average network, each node is assigned a score of one each time 1) the node is in the top 25% of nodes with strongest $S_{i}$, or 2) it is placed in the top 25% of nodes with highest $B_{i}$, or 3) it is in the top 25% of nodes with highest $E_{i}$. With a maximum score of three, nodes that score two or higher are classified as hubs. The threshold for assigning a hubness score, although similar to previous work van den Heuvel et al. (2010), is arbitrary. Therefore, we investigate the extent to which the identified hubs in each group remain comparable while changing threshold cut from 10% to 50%. Fig. 2 displays a toy network where regions with high $S_{i}$, $E_{i}$, and $B_{i}$ are defined as hubs. In this work, we refer to the set of edges connecting the hubs as the hub sub-network.

### 2.9 Consensus classification of peripheral regions

Peripheral nodes present low $B_{i}$, $S_{i}$ and $E_{i}$. Based on each measure, a region is assigned a score of one if it is in the 25% of nodes with lowest $S_{i}$, $E_{i}$, or $B_{i}$. With a maximum score of three, we label regions with scores of two or above as peripheral regions. As mentioned for the identification of hub regions, the threshold of 25% is arbitrary. Therefore, we examine comparability of the peripheral regions for EP and FT by changing threshold cut from 10% to 50%. Fig. 2 shows a toy network where regions with low $S_{i}$, $E_{i}$, and $B_{i}$ are characterised as peripheral. The set of edges connecting the peripheral nodes are referred to as peripheral sub-network.

### 2.10 Microstructural features

Using the microstructural networks derived in Section 2.5, we examine FA, MD, NDI, and ODI properties of the identified hub and peripheral sub-networks between EP and FT. We investigate the link between the pattern of microstructural connectivity and the gestational age of EP. A GLM with TBV and group membership as covariates is fitted to each microstructural property. Moreover, EP are split into three groups according to their gestational age at birth (see Table 1) and compared in terms of their measures of microstructural parameters. The analysis is conducted across both the WM of the hub and the peripheral sub-networks. We analysed the group mean differences by running Kruskal-Wallis statistical test. We reported the mean and computed the confidence intervals for populations differences using the approach suggested by Campbell and Gardner (1988). The inferences are corrected for multiple comparisons (Bonferroni correction).

## 3 Results

### 3.1 Statistical analysis

**Fig. 3:**
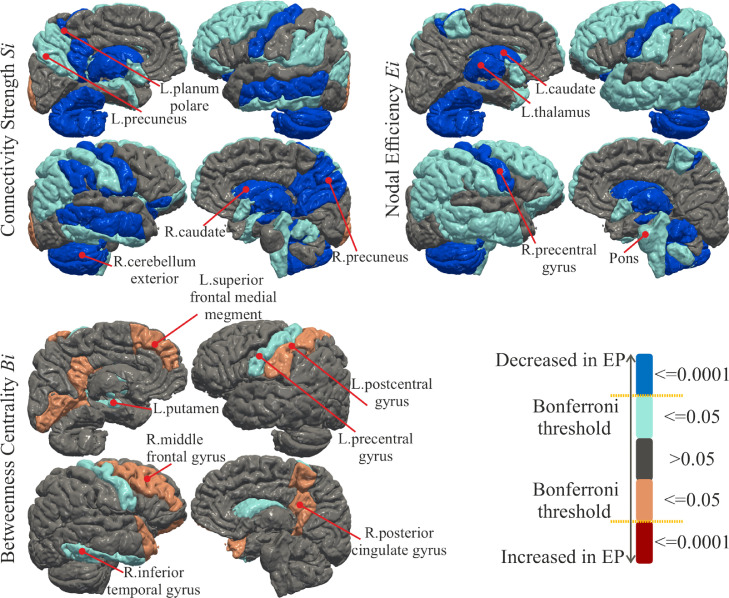
Statistical comparison using student’s *t*-test between the Full-Term (FT) and Extremely Preterm (EP) subjects on node-specific *Connectivity Strength*$S_{i}$, *Nodal Efficiency*$E_{i}$ and *Betweenness Centrality*$B_{i}$. The measures in regions in shades of red colours are significantly increased in the EP born subjects, while the measures in regions in shades of blue colours are significantly reduced in the EP born subjects. The regions in grey colour do not show a significant between-group difference. Darker shades indicate that the region is significantly different after Bonferroni correction (α=0.0001) and lighter colours indicate that the statistic in the region is below the standard threshold p=.05 but above the critical Bonferroni threshold.

**Fig. 4:**
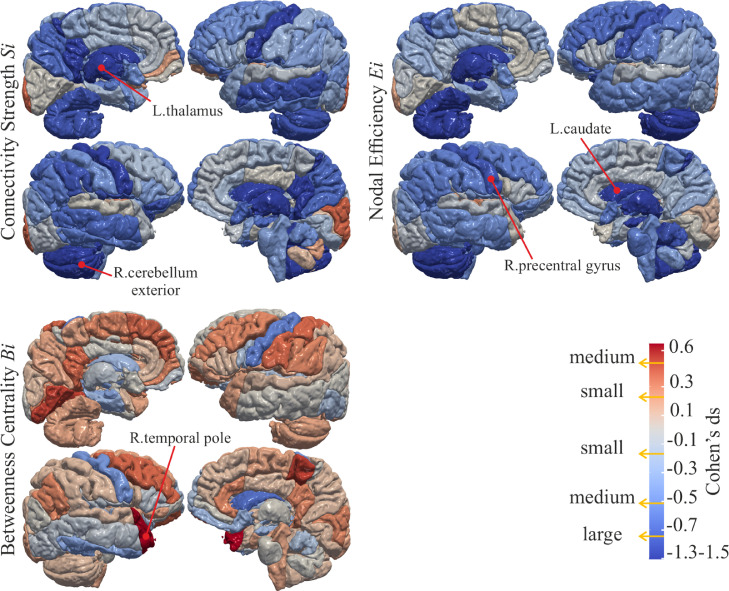
Maps of the effect size analysis of the difference between EP and FT on measures of *Connectivity Strength*$S_{i}$, *Nodal Efficiency*$E_{i}$ and *Betweenness Centrality*$B_{i}$. The regions coloured in shades of blue are the regions where EP have decreased the specific graph measure, while the regions in shades of red are where EP have increased the specific graph measure.

The analysis of $S_{i}$ reveals overall reduced strength of connectivity in the EP brain as shown in the top left of Fig. 3. Reductions are mainly driven by the cerebellum, deep nuclei, mid-temporal area, precentral gyrus and, precuneus, which also demonstrate the largest effect sizes (Fig. 4). The global connectivity S is significantly reduced ($p=6.5e^{-5}$) in EP (S=500±67) compared to FT (S=554±76). Similarly, the networks of EP show decreased $E_{i}$ in the cerebellum, deep nuclei, and precentral gyrus as illustrated in the top right of Fig. 3, which is in line with larger effect sizes in the same regions (Fig. 4). To a smaller extent the $E_{glob}$ is reduced ($p=9.8e^{-3}$) with $E_{glob}=0.15\pm 0.02$ in EP and $E_{glob}=0.16\pm 0.02$ in FT. The EP network reveals trends of reduced $B_{i}$ in the precentral, and increased $B_{i}$ in the middle frontal and postcentral gyri, especially medially. As displayed in the bottom of Fig. 3, the differences in $B_{i}$ are not significant after Bonferroni correction and the effect sizes are smaller (Fig. 4). In general, effect sizes are small to medium for $B_{i}$, with EP having larger $B_{i}$ than FT; effect sizes are small to large, with EP having reduced $S_{i}$ and $E_{i}$ than FT. Tables 1, 2, and 3 of the Supplementary Material (SM) report the means, statistics, and the effect size for each brain region.

**Fig. 5:**
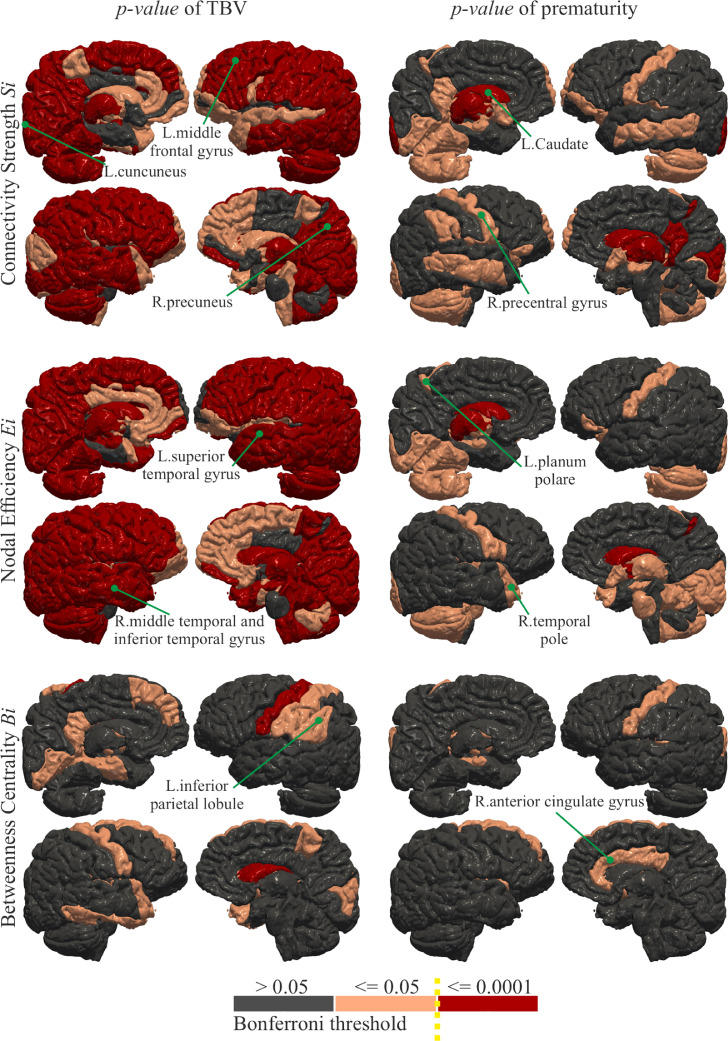
The significance maps of the effect of TBV and group membership on $S_{i}$, $E_{i}$, and $B_{i}$. The colour scale for statistical significance ranges from grey (node is not statistically significance), light red (node is significant at p−value lower than 0.05) to dark red (significant after Bonferroni correction).

**Fig. 6:**
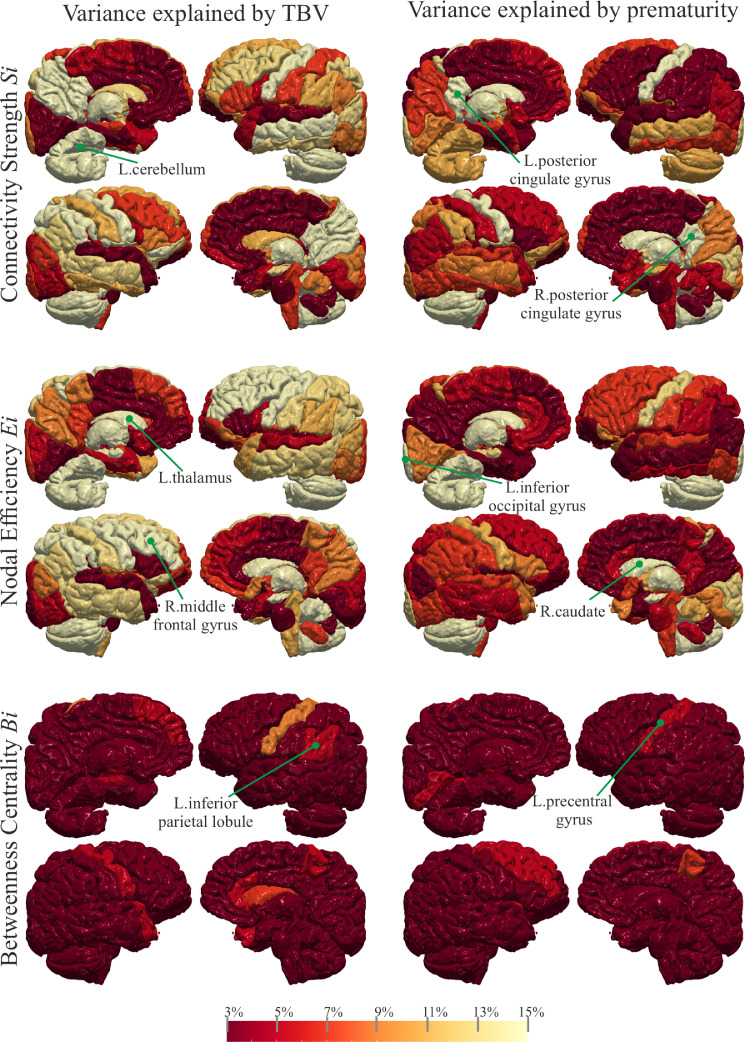
Maps of the variance explained by TBV and prematurity on $S_{i}$$E_{i}$ and $B_{i}$. The colour scale ranges from dark red, indicating that the regressor explains little variance (from 0 to 3%), to yellow indicating the regressors larger explain (from 13% to 15%).

The TBV of EP (μ=1019.7±23.4 cm${}^{3}$) is significantly ($p=8e^{-6}$) reduced compared to FT (μ=1112.0±30.4 cm${}^{3}$) (see Figure 1 of the SM ). The analysis of the covariates reveals that the regions with significantly reduced $S_{i}$ in EP are influenced both by TBV and premature birth (top of Fig. 5). The variation of $S_{i}$ in bilateral thalamus, bilateral putamen, bilateral caudate, left occipital pole, right pallidum, right post central gyrus medial segment, right posterior cingulate gyrus, and right cuneus is explained by premature birth. Correspondingly, bilateral thalamus, caudate, pallidum, and posterior cingulate gyrus show highest variances explained (Fig. 6). As illustrated in Fig. 5, the regions with significantly low $E_{i}$ in EP show both group and TBV effect, which is reflected in similar amounts of variances explained (Fig. 6). However, the reduction of $E_{i}$ in left thalamus, bilateral caudate, right pallidum, and post central gyrus medial segment is mainly explained by premature birth. The variation of $B_{i}$ (Fig. 5) in left precentral gyrus and right caudate is affected by being born extremely preterm. Much lower variance explained is observed accordingly (Fig. 6). There is no statistically significant difference in TBV, $S_{i}$, $E_{i}$, and $B_{i}$ between the subgroups (split by gestational age) of EP.

### 3.2 Consensus identification of hubs

**Fig. 7:**
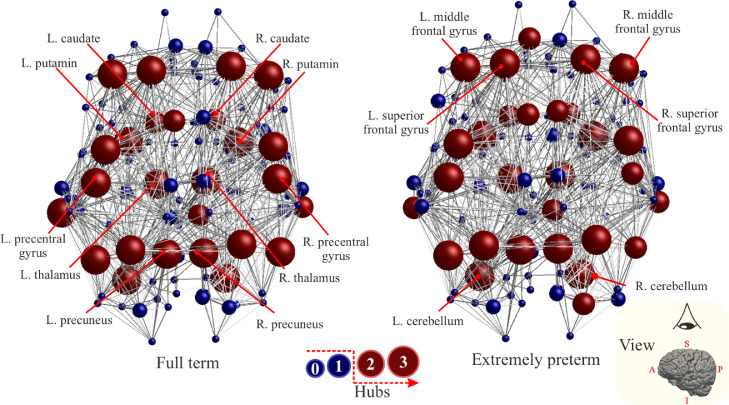
Node-specific hubness scores for the average brain network of Full-Term born subjects (FT) (left) and Extremely Preterm born subjects (EP) (right). Each node is assigned a score of one each time it is in the top 25% of nodes with highest $S_{i}$, $E_{i}$, and $B_{i}$. Node with scores equal or higher than two are classified as hub and are coloured in red. Regions with lower scores are coloured in blue.

The hubs identified for FT and EP are depicted in Fig. 7. Nodes in red are the brain regions that scored more or equal to two while nodes in blue are the brain regions that scored less or equal to one. Overall, 26 regions are identified for the FT, 29 for the EP, while the common regions are 26. Varying the threshold for identifying hubs results in an overlap, that ranges from 86%to100%, between the identified regions in both groups.

Left superior frontal gyrus medial segment, right supplementary motor cortex, and superior occipital gyrus are identified as hubs only in the EP group. The full list of the identified putative hubs is provided in Table 4 of the SM.

### 3.3 Consensus classification of peripheral regions

**Fig. 8:**
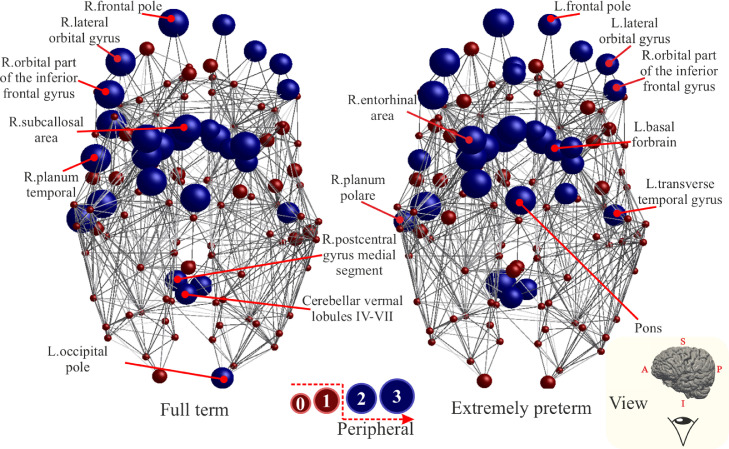
Node-specific peripheral scores for the average brain network of Full-Term born subjects (FT) (left) and Extremely Preterm born subjects (EP) (right). Each node is assigned a score of one each time it is in the bottom 25% of nodes with lowest $S_{i}$, $E_{i}$ and $B_{i}$. Node with scores equal or higher than 2 are classified as peripheral regions and are coloured in blue, nodes that scores less are coloured in red.

The regions identified as peripheral in the FT and EP average structural networks are illustrated in Fig. 8. Thirty-one regions are identified for the FT and 28 are identified for the EP; 27 of which are common between the two groups.

Anterior orbital gyrus and occipital pole are found only in the FT network, while gyrus rectus is found only in the EP network. The list of identified peripheral regions is provided in Table 5 of the SM.

### 3.4 Microstructural features

The results of the comparison between microstructural parameters of EP and FT is illustrated in Table 2. The table shows a global statistically significant lower FA and NDI and higher MD in EP. The analysis of the microstructural properties on the hub and peripheral sub-networks reveals a similar course. ODI is significantly higher in EP along the peripheral sub-network. Although FA, MD, and NDI show statistically significant difference between the groups, only FA and MD remain significant after Bonferroni correction with α=0.05/12 (four microstructural measures per three types of WM areas) in hubs sub-network ($p_{\text{FA}}=1.23e^{-3},p_{\text{MD}}=2.32e^{-5}$), peripheral sub-network ($p_{\text{FA}}=4.94e^{-4},p_{\text{MD}}=2.97e^{-5}$), and global connectivity ($p_{\text{FA}}=2.60e^{-4},p_{\text{MD}}=5.13e^{-5}$) (see Table 2).

**Table 2:** Comparison between Extremely Preterm born subjects (EP) and the Full-Term born subjects (FT) on the microstructural properties in hub and peripheral sub-networks, and global connectivity. The statistical analysis is performed using Kruskal-Wallis statistical test; The p-values in bold are statistically significant, and the ones marked by an asterisk are statistically significant after Bonferroni correction with α=0.004. IQR refers to the interquartile range of the data, and 95% CI is the 95% confidence interval for the difference in the median.

FA	FT median (IQR)	EP median (IQR)	p-value	95% CI
Hubs sub-network	0.472(0.020)	0.462(0.026)	$1.23e^{-3*}$	$\left(4.47e^{-3},\;1.81e^{-2}\right)$
Peripheral sub-network	0.434(0.026)	0.420(0.029)	$4.94e^{-4*}$	$\left(6.59e^{-3},\;2.06e^{-2}\right)$
Global connectivity	0.461(0.021)	0.447(0.025)	$2.60e^{-4*}$	$\left(6.09e^{-3},\;2.02e^{-2}\right)$
MD $*1e^{-3}\left[\text{mm}{\$}^{2}\$/s\right]$				
Hubs sub-network	0.665(0.031)	0.685(0.041)	$2.32e^{-5*}$	$\left(-2.93e^{-2},\;-1.13e^{-2}\right)$
Peripheral sub-network	0.669(0.028)	0.688(0.043)	$2.97e^{-5*}$	$\left(-2.66e^{-2},\;-9.33e^{-3}\right)$
Global connectivity	0.668(0.028)	0.687(0.044)	$5.13e^{-5*}$	$\left(-2.70e^{-2},\;-9.33e^{-3}\right)$
NDI				
Hubs sub-network	0.600(0.035)	0.584(0.040)	$1.85e^{-2}$	$\left(2.03e^{-3},\;2.26e^{-2}\right)$
Peripheral sub-network	0.577(0.031)	0.562(0.038)	$1.04e^{-2}$	$\left(2.95e^{-3},\;2.27e^{-2}\right)$
Global connectivity	0.592(0.036)	0.576(0.040)	$1.41e^{-2}$	$\left(2.65e^{-3},\;2.27e^{-2}\right)$
ODI				
Hubs sub-network	0.257(0.010)	0.258(0.011)	$4.74e^{-1}$	$\left(-3.96e^{-3},\;1.90e^{-3}\right)$
Peripheral sub-network	0.279(0.014)	0.282(0.015)	$3.00e^{-2}$	$\left(-7.80e^{-3},\;-3.82e^{-4}\right)$
Global connectivity	0.265(0.010)	0.266(0.012)	$9.08e^{-2}$	$\left(-5.62e^{-3},\;3.44e^{-4}\right)$

Table 2 reports the 95% confidence intervals for the difference of medians between EP and FT. Values are at least one order of magnitude lower than the values of the parameters, indicating that the difference, although statistically significant, is small. The analysis of the covariates shows that TBV drives the variations in FA and ODI. The variance explained by TBV is around 22% and 40% , respectively, for all the three brain areas. Extreme preterm birth affects the differences in MD (around 11% of variance explained) and to a lesser extent, the differences in NDI (around 3% of variance explained). The details about this analysis are in Table 6 of the SM.

Table 7 of the SM reports the results when comparing the microstructural parameters at three gestational groups (refer to Table 1) of the EP. Overall, there is no evidence to suggest that there is significant change in microstructural parameters with respect to gestational age. However, compared to subjects born at 25 completed weeks of gestation, subjects born at 24 completed weeks of gestation have statistically significant lower FA in the hubs sub-network (p=.021;95%=(−0.025,−0.002)) and in peripheral sub-network (p=.014;95%=(−0.025,−0.003)), and have a statistically significant higher ODI in the hubs sub-network (p=.014;95%=(0.001,0.011)) and in peripheral sub-network (p=.003;95%=(0.003,0.015)). None of these tests are significant after multiple comparison correction (Bonferroni α=0.002).

## 4 Discussion

In this work, we analyse the network structure of a large single-site population of extremely preterm and full-term born young adults to evaluate the long-term influence of prematurity on brain architecture and microstructure. Further, we examine the link between the degree of prematurity and the extent of microstructural change. The study is carried out by combining graph theory measures Rubinov and Sporns (2010) with the state-of-the-art models for WM structure Tournier et al. (2007) and microstructure estimation Basser et al. (1994); Zhang et al. (2012).

We investigate several features of brain networks; of these, *nodal efficiency* $E_{i}$ indicates how closely nodes are connected to other nodes in the network, with shorter paths conveying information flow more efficiently Latora and Marchiori (2001). The reduced $E_{i}$ in EP (Fig. 3 top right) might suggest that the integration of information flow in the connections to precentral gyrus, deep GM regions, and cerebellum exterior is altered in EP. Moreover, the strength of connectivity $S_{i}$ (Fig. 3 top left) in the same areas of the brain is significantly diminished in EP ; alterations in the same areas are reported by previous studies in preterm infants Ball et al. (2013a) and young adults Irzan, Fidon, Vercauteren, Ourselin, Marlow, Melbourne, 2020, Irzan, Hütel, Semedo, O’Reilly, Sahota, Ourselin, Marlow, Melbourne, 2020. Our results suggest that the connectivity alterations after preterm birth do not change substantially from infancy to 19-years of age.

Furthermore, the analysis suggests that reduced connectivity $S_{i}$ is implicated in the decrease of $E_{i}$. However, the reduction of the global connectivity S is not associated with as strong a reduction in *global efficiency* $E_{glob}$. This result might imply a localised effect of prematurity restricted to specific regions . Results of decreased connectivity strength and global efficiency after preterm birth are reported by previous studies de Almeida et al. (2021); Batalle et al. (2017). The finding of lower $B_{i}$ in EP in left putamen, left hippocampus, right caudate, right inferior temporal gyrus, and bilateral precentral gyrus suggests a less central role of these regions. All these regions relate to the brain areas of somato-sensory function. In a typical pregnancy, the primary somato-sensory functions are developed inside the mother’s womb during the third trimester of pregnancy Silbereis et al. (2016). As the extremely preterm born individuals develop *ex utero*, these functions have to be refined after birth in the extrauterine environment and this might have induced differences. At the same time, the finding of a higher $B_{i}$ in left superior frontal gyrus medial segment, bilateral posterior cingulate gyrus, left calcarine cortex, left lingual gyrus, right medial orbital gyrus, right middle frontal gyrus, right precentral gyrus medial segment, and right temporal pole shows higher centrality of these areas in the EP brain. As these regions undergo late-maturation, our results might suggest a long-term plasticity that allowed to reinforce those regions to compensate for the regions of lower $B_{i}$. A compensatory mechanism might have driven the increased volume of these regions as suggested by a previous study on this dataset (Irzan, O’Reilly, Ourselin, Marlow, Melbourne, 2020) . Although the impact of these results remain unclear, the findings suggest possible compensatory processes to alleviate the effects of premature birth.

The conducted ANOVA shows that the reduced TBV in EP (Figure 1 of the SM) is principally implicated in inducing lower $S_{i}$, $E_{i}$, and $B_{i}$. To a minor extent, the prematurity has significant impact too (see Fig. 6). The TBV is significantly related to the connectivity of all the cerebral cortex regions as well as to the deep WM regions, while the prematurity appears to explain mainly the changes in connections to the deep GM regions and somato-sensory areas.

The consensus identification of hubs aims to capture distinct aspects of topological paths centrality of the putative hub regions: $S_{i}$ captures the centrality of nodes that have multiple and strong connections with the assumption that these exert more influence on brain function. Under the hypothesis that the information travels through the brain'snetwork *via* the shortest path, $B_{i}$ describes the centrality of nodes that lie on many shortest path and hence mediate high proportion of traffic information Freeman (1977). $E_{i}$ Latora and Marchiori (2001) describes the centrality of a node with the intuition that a node with a short average path length mediates network integration and delivery of information more efficiently. Therefore, the identified putative hubs have strong connections to many other nodes and mediate traffic information with other nodes *via* shortest paths. The regions with a hub score of three that are common between the groups included bilateral thalamus, putamen, precuneus, middle frontal gyrus, superior parietal lobule, angular gyrus, precuneus, precentral gyrus, superior frontal gyrus, and cerebellum exterior. The results are similar to previous findings on healthy subjects (van den Heuvel, Mandl, Stam, Kahn, Pol, 2010). The comparable hub architecture between EP and FT suggests that the establishment of such architecture is perhaps minimally affected by the premature exposure to the extrauterine conditions. This confirms the result of earlier studies in which the authors found that there is an intact densely connected hub architecture in preterm born infants, concluding that such architecture might be a property of complex brain networks (Ball, Aljabar, Zebari, Tusor, Arichi, Merchant, Robinson, Ogundipe, Rueckert, Edwards, Counsell, 2014).

The overall trend suggested by Table 2 is that the FA and NDI are higher in hub sub-network than in peripheral sub-network, while MD and ODI are higher in peripheral sub-network than in hub sub-network. This is consistent with our definition of hubs and peripheral connectivity, as the hubs are the areas of the brain of packed WM fibre bundles which are more scarce in the peripheral areas.

We analyse the microstructural features of the networks and find that EP exhibit significant lower FA in hub and peripheral sub-networks as well as across the entire brain connectivity compared to their FT equivalent (Table 2). This finding suggests that the WM connections to the hub regions of the brain, such as deep GM regions and regions in the frontal cortex, are developmentally altered after preterm birth. Several previous studies report lower FA in preterm adolescents Allin et al. (2011); Eikenes et al. (2011); Fischi-Gómez et al. (2015) in major WM tracts passing near the thalamus Eikenes et al. (2011) and overall reduction FA Fischi-Gómez et al. (2015). Due to the lack of specificity of DTI, changes in FA can result from changes in several WM microstructural properties such as axonal density, axon orientation dispersion, and myelination Jones and Cercignani (2010). The NODDI model Zhang et al. (2012) aims to estimate the axonal density (NDI) and the axonal orientation dispersion (ODI) separately and hence can disentangle two fundamental contributors to the FA parameter Colgan et al. (2016). In the present analysis, axonal density is significantly lower in EP along WM tracts of hub sub-network, peripheral sub-network and over the overall networks (Table 2), suggesting that the low FA in EP might be due to a reduction in axonal density (NDI). The comparable values for ODI between EP and FT might suggest that the spatial configuration of the neurites structures is comparable between the two groups. Furthermore, as shown in Table 2 there is significantly higher MD in the hub, the peripheral sub-network, and the global connectivity of EP. Higher MD and lower NDI might indicate a loss in the neurite density. As observed by earlier studies, we did not identify any brain areas where prematurity is linked to an increase in either FA or NDI or a decrease in either ODI or MD parameters Batalle et al. (2017).

The statistical comparison of microstructural parameters in hub and peripheral sub-networks between EP sub-groups reveals that the main differences are observed when comparing FA and ODI of subjects born at 24 and 25 weeks of gestation: lower FA and higher ODI with decreasing gestational age (see Table 7 of the SM). Although the changes are not statistically significant after correcting for multiple comparison, the trend suggests that the degree of alteration of the microstructural properties might be linked to increased prematurity. It is hard to determine whether such a result is due to decreased sample size, or it is plausible that at this age (19 years) there are no microstructural differences between subjects born extremely preterm.

The comparable hub topology between the groups may suggest that even though hubs promote functional integration processes, they do not determine their outcome, which instead might be found in the overall dynamics of the brain. Although the hubs architecture appears to remain intact, there is evidence of a significant alteration of WM connectivity at both the macro- and microstructural level. It is important to highlight that many regions (such deep GM and frontal connections) in the hub sub-network are found to have altered $S_{i}$ and $E_{i}$ in EP. This provides further evidence that WM abnormalities associated with the premature exposure to the extrauterine environment are present not only at term equivalent age Ball et al. (2013b) but persist into early adulthood.

## 5 Technical considerations

In our statistical analysis, we include TBV and group membership (EP vs FT) as covariates. We do not include sex as a regressor because, from previous analysis Irzan, Marlow, Ourselin, Melbourne, 2020, Irzan, O’Reilly, Ourselin, Marlow, Melbourne, 2020; O’Reilly et al. (2020) on this dataset, we have little evidence that there is significant difference between female and male subjects. Moreover, additional regressors would reduce the statistical power of the analysis.

To test the hypothesis that the volume of ROI would explain some variance of the graph measures, we add one further regressor to account for the volume of ROI in the GLM. We find that such an extended model has a larger standard error and cannot provide a clear cut in disentangling the effect of TBV and the volume of ROI. Section 3 of the SM describes in detail the results of the analysis.

The interpretation of the statistical results heavily depends on the method employed for adjusting for multiple comparisons and the level of significance set. In order to correct for type I error due to the significant number of statistical tests conducted, we use Bonferroni correction. Other studies adopt other techniques such as false discovery rate Genovese et al. (2002). Unfortunately, there is no unanimous consensus on which approach is the most suitable; which may lead to a considerable variation in the results. We are conscious that our approach is conservative with the risk of increasing false negatives; however, we are more confident about the plausibility of our findings to be true positives.

The graph theory measure which best describes the flow of information in the brain is unclear Fornito et al. (2016), and at present, nodal efficiency relies on the assumption that each brain region has global knowledge of the architecture of the brain-network Fornito et al. (2016), which is hard to test. Moreover, the structural network is far removed from the complexity of the rapid biological interactions at the axon level, and the flow of information in the ‘static’ structural network is a rather coarse way of encoding the information flow in a dynamic system such as the brain.

The results provide an overview of the impact of extreme prematurity on highly functioning subjects who have completed upper secondary education and are not severely disabled. This analysis could therefore give a more optimistic view of the potential outcome of extremely premature birth. This study might also suffer from possible bias effects due to the typical consequence of monocentric study such as centre, scanner, recruitment, and geographical area; however, the present analysis was carried out on a large single-site population of 19-year old young adults with no differences in age, socioeconomic status, and quality of care. These characteristics of the data allow the analysis to rule out some hidden factors that affect other studies such as MRI scanner, and age at scan. Furthermore, the volume of brain structures and brain networks are measured in the subject’s native space , reducing from the workflow additional uncertainty due to registration error.

## 6 Conclusions and future work

In this study of 19-year-old young adults born before 26 weeks of gestation, we have evaluated the long-term effects of extreme preterm birth on brain network characteristics against a control population. Although the hierarchical structure of the extremely preterm brain is preserved, it has significantly less structural connectivity and tissue cellularity, and the most significant alterations are observed in the connections between the central areas of the brain. This MRI-based phenotype is important and allows us to investigate performance of the cohort in relation to our findings in future studies and help us better predict long-term cognitive outcomes for extremely preterm born babies born today.

## Credit Author Statment

Hassna Irzan conceived and conducted the analysis, drafted the manuscript and revised it.

Erika Molteni contributed to interpreting the results and contributed to revisions.

Michael Hütel contributed to the initial discussion and interpretation of the data, and revised the final manuscript.

Sebastien Ourselin contributed to funding and supervision.

Neil Marlow initiated the project, secured the funding, and supervised the project.

Andrew Melbourne collected the data, discussed the results, and revised the final manuscript.

## Data and code availability statement

The data of this study are obtained from the EPICure project. Due to restrictions imposed by the administering institution, the data is not openly available. We provide detailed tables in the supplementary material reporting the intermediate steps of the analysis.

The code that supports the findings of this study is available from the corresponding author.

## Appendix A Supplementary materials

Supplementary Data S1 Supplementary Raw Research Data. This is open data under the CC BY license http://creativecommons.org/licenses/by/4.0/ Supplementary Data S1

## References

[bib0001] Allin M.P., Kontis D., Walshe M., Wyatt J., Barker G.J., Kanaan R.A., McGuire P., Rifkin L., Murray R.M., Nosarti C. (2011). White matter and cognition in adults who were born preterm. PLoS ONE.

[bib0002] de Almeida J.S., Meskaldji D.-E., Loukas S., Lordier L., Gui L., Lazeyras F., Hüppi P.S. (2021). Preterm birth leads to impaired rich-club organization and fronto-paralimbic/limbic structural connectivity in newborns. Neuroimage.

[bib0003] Andersson J.L., Sotiropoulos S.N. (2016). An integrated approach to correction for off-resonance effects and subject movement in diffusion mr imaging. Neuroimage.

[bib0004] Ball G., Aljabar P., Zebari S., Tusor N., Arichi T., Merchant N., Robinson E.C., Ogundipe E., Rueckert D., Edwards A.D., Counsell S.J. (2014). Rich-club organization of the newborn human brain. Proceedings of the National Academy of Sciences.

[bib0005] Ball G., Boardman J.P., Aljabar P., Pandit A., Arichi T., Merchant N., Rueckert D., Edwards A.D., Counsell S.J. (2013). The influence of preterm birth on the developing thalamocortical connectome. Cortex.

[bib0006] Ball G., Boardman J.P., Rueckert D., Aljabar P., Arichi T., Merchant N., Gousias I.S., Edwards A.D., Counsell S.J. (2012). The effect of preterm birth on thalamic and cortical development. Cereb. Cortex.

[bib0007] Ball G., Pazderova L., Chew A., Tusor N., Merchant N., Arichi T., Allsop J.M., Cowan F.M., Edwards A.D., Counsell S.J. (2015). Thalamocortical connectivity predicts cognition in children born preterm. Cerebral cortex.

[bib0008] Ball G., Srinivasan L., Aljabar P., Counsell S.J., Durighel G., Hajnal J.V., Rutherford M.A., Edwards A.D. (2013). Development of cortical microstructure in the preterm human brain. Proceedings of the National Academy of Sciences.

[bib0009] Basser P.J., Mattiello J., LeBihan D. (1994). Mr diffusion tensor spectroscopy and imaging. Biophys. J..

[bib0010] Batalle D., Hughes E.J., Zhang H., Tournier J.-D., Tusor N., Aljabar P., Wali L., Alexander D.C., Hajnal J.V., Nosarti C. (2017). Early development of structural networks and the impact of prematurity on brain connectivity. Neuroimage.

[bib0011] Brown C.J., Miller S.P., Booth B.G., Andrews S., Chau V., Poskitt K.J., Hamarneh G. (2014). Structural network analysis of brain development in young preterm neonates. Neuroimage.

[bib0012] Bullmore E., Sporns O. (2009). Complex brain networks: graph theoretical analysis of structural and functional systems. Nat. Rev. Neurosci..

[bib0013] Campbell M.J., Gardner M.J. (1988). Statistics in medicine: calculating confidence intervals for some non-parametric analyses. Br. Med. J. (Clin Res Ed).

[bib0014] Cardoso M.J., Modat M., Wolz R., Melbourne A., Cash D., Rueckert D., Ourselin S. (2015). Geodesic information flows: spatially-variant graphs and their application to segmentation and fusion. IEEE Trans. Med. Imaging.

[bib0015] Colgan N., Siow B., O’Callaghan J.M., Harrison I.F., Wells J.A., Holmes H.E., Ismail O., Richardson S., Alexander D.C., Collins E.C., Fisher E.M.C., Johnson R.A., Schwarz A.J., Ahmed Z., O’Neill M.J., Murray T.K., Zhang H., X L.M.F. (2016). Application of neurite orientation dispersion and density imaging (noddi) to a tau pathology model of alzheimer’s disease. Neuroimage.

[bib0016] Costeloe K.L., Hennessy E.M., Haider S., Stacey F., Marlow N., Draper E.S. (2012). Short term outcomes after extreme preterm birth in england: comparison of two birth cohorts in 1995 and 2006 (the epicure studies). BMJ : British Medical Journal.

[bib0017] Eaton-Rosen Z., Melbourne A., Orasanu E., Beckmann J., Stevens N., Atkinson D., Marlow N., Ourselin S. (2016). White matter alterations in young adults born extremely preterm: a microstructural point of views. Proc. 24${}^{t}h$ Annual meeting of the international society for magnetic resonance in medicine - ismrm (Singapore, May 2016).

[bib0018] Eikenes L., Løhaugen G.C., Brubakk A.-M., Skranes J., Håberg A.K. (2011). Young adults born preterm with very low birth weight demonstrate widespread white matter alterations on brain dti. Neuroimage.

[bib0019] Fischi-Gómez E., Vasung L., Meskaldji D.-E., Lazeyras F., Borradori-Tolsa C., Hagmann P., Barisnikov K., Thiran J.-P., Hüppi P.S. (2015). Structural brain connectivity in school-age preterm infants provides evidence for impaired networks relevant for higher order cognitive skills and social cognition. Cerebral Cortex.

[bib0020] Fornito A., Zalesky A., Bullmore E. (2016). Fundamentals of brain network analysis.

[bib0021] Freeman L.C. (1977). A set of measures of centrality based on betweenness. Sociometry.

[bib0022] Frey H.A., Klebanoff M.A. (2016). The epidemiology, etiology, and costs of preterm birth. Seminars in Fetal and Neonatal Medicine.

[bib0023] Genovese C.R., Lazar N.A., Nichols T. (2002). Thresholding of statistical maps in functional neuroimaging using the false discovery rate. Neuroimage.

[bib0024] Hedderich D.M., Bäuml J.G., Menegaux A., Avram M., Daamen M., Zimmer C., Bartmann P., Scheef L., Boecker H., Wolke D. (2020). An analysis of mri derived cortical complexity in premature-born adults: regional patterns, risk factors, and potential significance. Neuroimage.

[bib0025] van den Heuvel M.P., Mandl R.C., Stam C.J., Kahn R.S., Pol H.E.H. (2010). Aberrant frontal and temporal complex network structure in schizophrenia: a graph theoretical analysis. J. Neurosci..

[bib0026] van den Heuvel M.P., Sporns O. (2013). Network hubs in the human brain. Trends Cogn. Sci. (Regul. Ed.).

[bib0027] Irzan H., Fidon L., Vercauteren T., Ourselin S., Marlow N., Melbourne A. (2020). Min-cut Max-flow for Network Abnormality Detection: Application to Preterm Birth. Uncertainty for Safe Utilization of Machine Learning in Medical Imaging, and Graphs in Biomedical Image Analysis.

[bib0028] Irzan H., Hütel M., Semedo C., O’Reilly H., Sahota M., Ourselin S., Marlow N., Melbourne A. (2020). A Network-based Analysis of the Preterm Adolescent Brain Using Pca and Graph Theory. Computational Diffusion MRI.

[bib0029] Irzan H., Marlow N., Ourselin S., Melbourne A. (2020). Preterm adolescents have altered hub regions. Proc. 28${}^{t}h$ Annual meeting of the international society for magnetic resonance in medicine - ismrm (Online, August 2020).

[bib0030] Irzan H., O’Reilly H., Ourselin S., Marlow N., Melbourne A. (2020). Brain Volume and Neuropsychological Differences in Extremely Preterm Adolescents. Medical Ultrasound, and Preterm, Perinatal and Paediatric Image Analysis.

[bib0031] Jeurissen B., Tournier J.-D., Dhollander T., Connelly A., Sijbers J. (2014). Multi-tissue constrained spherical deconvolution for improved analysis of multi-shell diffusion mri data. Neuroimage.

[bib0032] Jones D.K., Cercignani M. (2010). Twenty-five pitfalls in the analysis of diffusion mri data. NMR Biomed..

[bib0033] Karolis V.R., Froudist-Walsh S., Brittain P.J., Kroll J., Ball G., Edwards A.D., Dell’Acqua F., Williams S.C., Murray R.M., Nosarti C. (2016). Reinforcement of the brain’s rich-club architecture following early neurodevelopmental disruption caused by very preterm birth. Cerebral Cortex.

[bib0034] Kellner E., Dhital B., Kiselev V.G., Reisert M. (2016). Gibbs-ringing artifact removal based on local subvoxel-shifts. Magn. Reson. Med..

[bib0035] Latora V., Marchiori M. (2001). Efficient behavior of small-world networks. Phys. Rev. Lett..

[bib0036] Marlow N., Wolke D., Bracewell M.A., Samara M. (2005). Neurologic and developmental disability at six years of age after extremely preterm birth. N top N. Engl. J. Med..

[bib0037] Moore T., Hennessy E.M., Myles J., Johnson S.J., Draper E.S., Costeloe K.L., Marlow N. (2012). Neurological and developmental outcome in extremely preterm children born in england in 1995 and 2006: the epicure studies. Br. Med. J..

[bib0038] O’Reilly H., Johnson S., Ni Y., Wolke D., Marlow N. (2020). Neuropsychological outcomes at 19 years of age following extremely preterm birth. Pediatrics.

[bib0039] Organization, W. H., 2012. Born too soon: the global action report on preterm birth.

[bib0040] Rubinov M., Sporns O. (2010). Complex network measures of brain connectivity: uses and interpretations. Neuroimage.

[bib0041] Saigal S., Doyle L.W. (2008). An overview of mortality and sequelae of preterm birth from infancy to adulthood. Lancet.

[bib0042] Silbereis J.C., Pochareddy S., Zhu Y., Li M., Sestan N. (2016). The cellular and molecular landscapes of the developing human central nervous system. Neuron.

[bib0043] Smith R.E., Tournier J.-D., Calamante F., Connelly A. (2012). Anatomically-constrained tractography: improved diffusion mri streamlines tractography through effective use of anatomical information. Neuroimage.

[bib0044] Smith R.E., Tournier J.-D., Calamante F., Connelly A. (2015). Sift2: enabling dense quantitative assessment of brain white matter connectivity using streamlines tractography. Neuroimage.

[bib0045] Sølsnes A.E., Sripada K., Yendiki A., Bjuland K.J., Østgård H.F., Aanes S., Grunewaldt K.H., Løhaugen G.C., Eikenes L., Håberg A.K. (2016). Limited microstructural and connectivity deficits despite subcortical volume reductions in school-aged children born preterm with very low birth weight. Neuroimage.

[bib0046] Tournier J.-D., Calamante F., Connelly A. (2007). Robust determination of the fibre orientation distribution in diffusion mri: non-negativity constrained super-resolved spherical deconvolution. Neuroimage.

[bib0047] Tournier J.-D., Smith R., Raffelt D., Tabbara R., Dhollander T., Pietsch M., Christiaens D., Jeurissen B., Yeh C.-H., Connelly A. (2019). Mrtrix3: a fast, flexible and open software framework for medical image processing and visualisation. Neuroimage.

[bib0048] Tustison N.J., Avants B.B., Cook P.A., Zheng Y., Egan A., Yushkevich P.A., Gee J.C. (2010). N4itk: Improved n3 bias correction. IEEE Trans. Med. Imaging.

[bib0049] Van Den Heuvel M.P., Kersbergen K.J., De Reus M.A., Keunen K., Kahn R.S., Groenendaal F., De Vries L.S., Benders M.J. (2015). The neonatal connectome during preterm brain development. Cerebral cortex.

[bib0050] Veraart J., Novikov D.S., Christiaens D., Ades-Aron B., Sijbers J., Fieremans E. (2016). Denoising of diffusion mri using random matrix theory. Neuroimage.

[bib0051] Young J.M., Vandewouw M.M., Mossad S.I., Morgan B.R., Lee W., Smith M.L., Sled J.G., Taylor M.J. (2019). White matter microstructural differences identified using multi-shell diffusion imaging in six-year-old children born very preterm. NeuroImage: Clinical.

[bib0052] Zhang H., Schneider T., Wheeler-Kingshott C.A., Alexander D.C. (2012). Noddi: practical in vivo neurite orientation dispersion and density imaging of the human brain. Neuroimage.

[bib0053] Zhao T., Mishra V., Jeon T., Ouyang M., Peng Q., Chalak L., Wisnowski J.L., Heyne R., Rollins N., Shu N. (2019). Structural network maturation of the preterm human brain. Neuroimage.

