# Supplementary material for: White matter analysis of the extremely preterm born adult brain
Source: Neuroimage. 2021 Aug 15;237:118112. doi: 10.1016/j.neuroimage.2021.118112 (PMC8285592; doi:10.1016/j.neuroimage.2021.118112)
Supplement: Supplementary Data S1 — Supplementary Raw Research Data. This is open data under the CC BY license http://creativecommons.org/licenses/by/4.0/ [file mmc1.pdf]

Supplementary material: White matter analysis of the extremely preterm born adult brain

1 Statistical analysis

Region	Mean EP	Mean FT	p-value	Cohen's ds
LeftAccumbensArea	0.4234	0.4771	0.0712	-0.3385
LeftThalamusProper	10.1199	12.5676	0.0000	-1.5130
LeftPutamen	9.3222	11.3945	0.0000	-1.4214
LeftPallidum	1.5598	1.8895	0.0000	-0.7977
LeftBasalForebrain	0.5794	0.7145	0.0027	-0.5733
LeftCaudate	7.2351	9.9441	0.0000	-1.2528
LeftAmygdala	0.7380	0.8925	0.0002	-0.7000
LeftHippocampus	4.1844	4.8739	0.0016	-0.5869
LeftMOrGmedialorbitalgyrus	3.8884	3.6489	0.2649	0.1937
LeftGRegyrusrectus	2.3473	2.3344	0.9287	0.0160
LeftTrIFGtriangularpartoftheinferiorfrontalgyrus	2.7932	3.0760	0.0714	-0.3328
LeftMFGmiddlefrontalgyrus	12.0229	13.0558	0.0075	-0.4907
LeftMFCmedialfrontalcortex	3.1609	2.9187	0.2295	0.2210
LeftOpIFGopercularpartoftheinferiorfrontalgyrus	4.0882	4.2308	0.3904	-0.1547
LeftLOrGlateralorbitalgyrus	1.4435	1.7663	0.0004	-0.6451
LeftOrIFGorbitalpartoftheinferiorfrontalgyrus	1.0398	1.2540	0.0005	-0.6126
LeftPOrGposteriororbitalgyrus	1.9244	2.2954	0.0001	-0.7339
LeftFRPfrontalpole	0.7579	0.9849	0.0015	-0.6163
LeftPrGprecentralgyrus	13.1314	14.9779	0.0000	-0.9128
LeftMPrGprecentralgyrusmedialsegment	4.2017	4.5065	0.1070	-0.3034
LeftCOcentraloperculum	3.2887	3.9130	0.0002	-0.6932
LeftSCAsubcallosalarea	0.5431	0.6408	0.0105	-0.4847
LeftSFGsuperiorfrontalgyrus	13.8155	15.2793	0.0021	-0.5611
LeftAOrGanteriororbitalgyrus	1.7617	1.9522	0.1085	-0.2868
LeftSMCsupplementarymotorcortex	6.3210	6.5954	0.2798	-0.1922
LeftFOfrontaloperculum	1.8556	2.0226	0.1753	-0.2458
LeftMSFGsuperiorfrontalgyrusmedialsegment	5.4069	5.4868	0.6894	-0.0727
LeftPPplanumpolare	2.4238	2.2541	0.3483	0.1597
LeftPTplanumtemporale	1.9633	2.2400	0.1126	-0.2917
LeftPHGparahippocampalgyrus	1.3406	1.4357	0.3175	-0.1787
LeftSTGsuperiortemporalgyrus	5.0149	5.0872	0.7435	-0.0583
LeftTMPtemporalpole	2.4949	2.7268	0.1117	-0.2928

LeftMTGmiddletemporalgyrus	7.3703	8.7109	0.0001	-0.7520
LeftTTGtransversetemporalgyrus	1.0201	1.1865	0.0192	-0.4427
LeftFuGfusiformgyrus	3.4332	3.6814	0.1241	-0.2947
LeftITGinferiortemporalgyrus	5.2083	6.0754	0.0012	-0.6228
LeftEntentorhinalarea	1.2465	1.4531	0.0085	-0.4615
LeftSPLsuperiorparietallobule	7.7709	8.4424	0.0133	-0.4534
LeftSMGsupramarginalgyrus	5.2297	5.7470	0.0198	-0.4349
LeftAnGangulargyrus	6.0676	6.4046	0.1134	-0.2925
LeftPCuprecuneus	7.2354	8.2386	0.0004	-0.6507
LeftPOparietaloperculum	2.1252	2.4760	0.0196	-0.4463
LeftPoGpostcentralgyrus	8.1469	8.5122	0.1750	-0.2512
LeftMPoGpostcentralgyrusmedialsegment	1.0978	1.5187	0.0000	-0.9030
LeftSOGsuperioroccipitalgyrus	5.1560	5.4315	0.1764	-0.2522
LeftCalccalcarinecortex	5.2778	5.2488	0.9109	0.0198
LeftCuncuneus	4.1396	4.1900	0.7920	-0.0487
LeftOCPoccipitalpole	2.3506	2.0548	0.0392	0.3668
LeftLiGlingualgyrus	3.9403	3.9664	0.8990	-0.0226
LeftOFuGoccipitalfusiformgyrus	4.0320	4.0703	0.8451	-0.0351
LeftIOGinferioroccipitalgyrus	5.3680	5.7211	0.1172	-0.2906
LeftMOGmiddleoccipitalgyrus	4.7164	5.2025	0.0061	-0.5035
LeftPCgGposteriorcingulategyrus	5.3629	6.4269	0.0000	-0.9991
LeftMCgGmiddlecingulategyrus	4.4355	4.7227	0.2838	-0.1938
LeftACgGanteriorcingulategyrus	4.1955	4.2571	0.7402	-0.0603
LeftPInsposteriorinsula	1.9871	2.1701	0.1785	-0.2625
LeftAInsanteriorinsula	2.8194	3.1140	0.0521	-0.3560
LeftCerebellumExterior	10.2575	11.5040	0.0000	-0.7864
RightAccumbensArea	0.4908	0.5212	0.2980	-0.1858
RightThalamusProper	10.0258	12.3329	0.0000	-1.3518
RightPutamen	8.7014	10.5403	0.0000	-1.1803
RightPallidum	1.4755	1.8403	0.0000	-0.9261
RightBasalForebrain	0.5094	0.6748	0.0001	-0.7548
RightCaudate	6.9748	9.8648	0.0000	-1.3574
RightAmygdala	0.5520	0.6571	0.0033	-0.5618
RightHippocampus	4.0777	4.9016	0.0001	-0.7385
RightMOrGmedialorbitalgyrus	3.9374	3.9866	0.8253	-0.0414
RightGRegyrusrectus	2.0322	2.1470	0.3381	-0.1684
RightTrIFGtriangularpartoftheinferiorfrontalgyrus	2.5804	2.8478	0.0505	-0.3704
RightMFGmiddlefrontalgyrus	11.4959	12.0457	0.1564	-0.2488
RightMFCmedialfrontalcortex	2.2940	2.4030	0.5024	-0.1219
RightOpIFGopercularpartoftheinferiorfrontalgyrus	3.8107	3.9737	0.2848	-0.1927
RightLOrGlateralorbitalgyrus	1.5890	1.9043	0.0005	-0.6397
RightOrIFGorbitalpartoftheinferiorfrontalgyrus	1.0092	1.2825	0.0001	-0.7977
RightPOrGposteriororbitalgyrus	1.9913	2.4474	0.0001	-0.7695
RightFRPfrontalpole	0.7611	1.0065	0.0009	-0.6181
RightPrGprecentralgyrus	12.1630	14.0481	0.0000	-0.9433
RightMPrGprecentralgyrusmedialsegment	4.0761	4.2052	0.4590	-0.1308
RightCOcentraloperculum	2.9461	3.7289	0.0000	-0.8270
RightSCAsubcallosalarea	0.4236	0.5368	0.0006	-0.6507

RightSFGsuperiorfrontalgyrus	14.0850	15.3935	0.0099	-0.4827
RightAOrGanteriororbitalgyrus	1.9759	2.1893	0.0697	-0.3429
RightSMCsupplementarymotorcortex	5.5496	5.7853	0.3045	-0.1910
RightFOfrontaloperculum	1.6243	2.0083	0.0009	-0.6307
RightMSFGsuperiorfrontalgyrusmedialsegment	5.3267	5.4826	0.4493	-0.1312
RightPPplanumpolare	1.1337	0.9473	0.1047	0.2544
RightPTplanumtemporale	1.2000	1.2178	0.8839	-0.0252
RightPHGparahippocampalgyrus	0.8487	0.8374	0.8494	0.0325
RightSTGsuperiortemporalgyrus	4.7192	4.7302	0.9533	-0.0107
RightTMPtemporalpole	1.9937	2.0500	0.6069	-0.0951
RightMTGmiddletemporalgyrus	5.9216	7.0293	0.0001	-0.7083
RightTTGtransversetemporalgyrus	0.7412	0.6994	0.4505	0.1373
RightFuGfusiformgyrus	2.8647	3.3362	0.0003	-0.6612
RightITGinferiortemporalgyrus	5.0180	5.7861	0.0021	-0.5791
RightEntentorhinalarea	0.9958	1.1395	0.0250	-0.3889
RightSPLsuperiorparietallobule	7.7703	8.6998	0.0002	-0.6953
RightSMGsupramarginalgyrus	4.2054	4.9915	0.0001	-0.7609
RightAnGangulargyrus	5.5953	6.2856	0.0026	-0.5520
RightPCuprecuneus	8.0832	9.3708	0.0001	-0.7101
RightPOparietaloperculum	2.0472	2.2090	0.2642	-0.1976
RightPoGpostcentralgyrus	6.7432	7.3884	0.0091	-0.4743
RightMPoGpostcentralgyrusmedialsegment	1.1056	1.6030	0.0000	-1.0935
RightSOGsuperioroccipitalgyrus	5.4809	5.7480	0.1968	-0.2218
RightCalccalcarinecortex	6.0793	5.8509	0.4278	0.1406
RightCuncuneus	4.8340	4.4160	0.0508	0.3529
RightOCPoccipitalpole	2.4594	2.1399	0.0283	0.3776
RightLiGlingualgyrus	4.3379	4.5483	0.3186	-0.1740
RightOFuGoccipitalfusiformgyrus	4.0439	4.1433	0.5670	-0.1021
RightIOGinferioroccipitalgyrus	5.5274	5.6778	0.4951	-0.1254
RightMOGmiddleoccipitalgyrus	3.4409	3.7789	0.0154	-0.4392
RightPCgGposteriorcingulategyrus	5.3761	6.4760	0.0000	-1.0572
RightMCgGmiddlecingulategyrus	4.2133	4.2308	0.9406	-0.0125
RightACgGanteriorcingulategyrus	3.2320	3.3881	0.2840	-0.1861
RightPInsposteriorinsula	2.1577	2.1161	0.6711	0.0723
RightAInsanteriorinsula	2.8840	3.1979	0.0375	-0.3864
RightCerebellumExterior	10.1600	11.6926	0.0000	-0.9974
Pons	0.1523	0.2012	0.0555	-0.3791
BrainStem	5.2133	5.6284	0.0305	-0.3997
CerebellarVermalLobulesI-V	4.0312	4.4010	0.0069	-0.4904
CerebellarVermalLobulesVI-VII	0.9941	0.9180	0.1802	0.2279
CerebellarVermalLobulesVIII-X	1.3462	1.2874	0.5182	0.1131

Table 1: Statics and effect size (Cohen's ds) of the comparison between Extremely Preterm born subjects (EP) and Full-Term born subjects (FT) on the *weighted connectivity strength* S_i of the structural brain networks. The brain regions are given by GIF parcellation.

Region	Mean EP	Mean FT	p-value	Cohen's ds
LeftAccumbensArea	0.0523	0.0566	0.1228	-0.2823
LeftThalamusProper	0.2207	0.2530	0.0000	-1.1147
LeftPutamen	0.2078	0.2332	0.0000	-0.8707
LeftPallidum	0.1122	0.1367	0.0000	-0.9653
LeftBasalForebrain	0.0671	0.0706	0.4174	-0.1440
LeftCaudate	0.2049	0.2418	0.0000	-1.0413
LeftAmygdala	0.0728	0.0783	0.0593	-0.3479
LeftHippocampus	0.1577	0.1836	0.0000	-0.8628
LeftMOrGmedialorbitalgyrus	0.1296	0.1346	0.3783	-0.1599
LeftGRegyrusrectus	0.1185	0.1205	0.6967	-0.0725
LeftTrIFGtriangularpartoftheinferiorfrontalgyrus	0.1484	0.1518	0.5923	-0.0991
LeftMFGmiddlefrontalgyrus	0.2494	0.2744	0.0006	-0.6520
LeftMFCmedialfrontalcortex	0.1332	0.1345	0.8324	-0.0395
LeftOpIFGopercularpartoftheinferiorfrontalgyrus	0.1795	0.1850	0.3132	-0.1834
LeftLOrGlateralorbitalgyrus	0.0912	0.1014	0.0230	-0.3970
LeftOrIFGorbitalpartoftheinferiorfrontalgyrus	0.0780	0.0894	0.0002	-0.6646
LeftPOrGposteriororbitalgyrus	0.1038	0.1214	0.0001	-0.7669
LeftFRPfrontalpole	0.0848	0.0997	0.0156	-0.4369
LeftPrGprecentralgyrus	0.2508	0.2799	0.0000	-0.8217
LeftMPrGprecentralgyrusmedialsegment	0.1970	0.2136	0.0079	-0.5085
LeftCOcentraloperculum	0.1631	0.1765	0.0299	-0.4144
LeftSCAsubcallosalarea	0.0679	0.0775	0.0118	-0.4739
LeftSFGsuperiorfrontalgyrus	0.2514	0.2759	0.0007	-0.6274
LeftAOrGanteriororbitalgyrus	0.1085	0.1129	0.4725	-0.1308
LeftSMCsupplementarymotorcortex	0.1977	0.1988	0.8570	-0.0328
LeftFOfrontaloperculum	0.1169	0.1196	0.5632	-0.1015
LeftMSFGsuperiorfrontalgyrusmedialsegment	0.1963	0.2061	0.1206	-0.2798
LeftPPplanumpolare	0.1226	0.1201	0.5977	0.0932
LeftPTplanumtemporale	0.1189	0.1208	0.7019	-0.0697
LeftPHGparahippocampalgyrus	0.1064	0.1159	0.0764	-0.3281
LeftSTGsuperiortemporalgyrus	0.1524	0.1551	0.5376	-0.1097
LeftTMPtemporalpole	0.1247	0.1350	0.0161	-0.4516
LeftMTGmiddletemporalgyrus	0.1806	0.1941	0.0113	-0.4707
LeftTTGtransversetemporalgyrus	0.0894	0.0904	0.8085	-0.0453
LeftFuGfusiformgyrus	0.1396	0.1475	0.0835	-0.3280
LeftITGinferiortemporalgyrus	0.1647	0.1798	0.0050	-0.5306
LeftEntentorhinalarea	0.0925	0.0994	0.0748	-0.3089
LeftSPLsuperiorparietallobule	0.2025	0.2190	0.0055	-0.5205
LeftSMGsupramarginalgyrus	0.1792	0.1890	0.0747	-0.3294
LeftAnGangulargyrus	0.1858	0.1981	0.0179	-0.4395
LeftPCuprecuneus	0.1927	0.2026	0.0812	-0.3105
LeftPOparietaloperculum	0.1242	0.1279	0.4443	-0.1398
LeftPoGpostcentralgyrus	0.2179	0.2370	0.0033	-0.5642
LeftMPoGpostcentralgyrusmedialsegment	0.1166	0.1414	0.0000	-0.7627

LeftSOGsuperioroccipitalgyrus	0.1542	0.1610	0.1423	-0.2668
LeftCalccalcarinecortex	0.1504	0.1513	0.8535	-0.0331
LeftCuncuneus	0.1478	0.1503	0.5959	-0.0967
LeftOCPoccipitalpole	0.1170	0.1132	0.3432	0.1725
LeftLiGlingualgyrus	0.1372	0.1379	0.8857	-0.0258
LeftOFuGoccipitalfusiformgyrus	0.1383	0.1433	0.2560	-0.2109
LeftIOGinferioroccipitalgyrus	0.1509	0.1573	0.1341	-0.2709
LeftMOGmiddleoccipitalgyrus	0.1545	0.1637	0.0470	-0.3604
LeftPCgGposteriorcingulategyrus	0.1806	0.1882	0.1689	-0.2489
LeftMCgGmiddlecingulategyrus	0.1659	0.1756	0.1521	-0.2623
LeftACgGanteriorcingulategyrus	0.1567	0.1579	0.8300	-0.0384
LeftPInsposteriorinsula	0.1192	0.1214	0.6429	-0.0882
LeftAInsanteriorinsula	0.1373	0.1524	0.0046	-0.5391
LeftCerebellumExterior	0.2259	0.2545	0.0000	-0.9326
RightAccumbensArea	0.0577	0.0594	0.4950	-0.1186
RightThalamusProper	0.2220	0.2530	0.0000	-0.9696
RightPutamen	0.2038	0.2271	0.0001	-0.7497
RightPallidum	0.1073	0.1331	0.0000	-0.9495
RightBasalForebrain	0.0640	0.0750	0.0151	-0.4394
RightCaudate	0.1972	0.2372	0.0000	-1.0862
RightAmygdala	0.0684	0.0709	0.3532	-0.1727
RightHippocampus	0.1585	0.1862	0.0000	-0.9267
RightMOrGmedialorbitalgyrus	0.1315	0.1380	0.2733	-0.2109
RightGRegyrusrectus	0.1108	0.1170	0.1817	-0.2493
RightTrIFGtriangularpartoftheinferiorfrontalgyrus	0.1421	0.1450	0.6271	-0.0905
RightMFGmiddlefrontalgyrus	0.2411	0.2629	0.0018	-0.5799
RightMFCmedialfrontalcortex	0.1200	0.1304	0.0980	-0.3137
RightOpIFGopercularpartoftheinferiorfrontalgyrus	0.1761	0.1775	0.7946	-0.0479
RightLOrGlateralorbitalgyrus	0.0970	0.1058	0.0407	-0.3556
RightOrIFGorbitalpartoftheinferiorfrontalgyrus	0.0788	0.0886	0.0035	-0.5388
RightPOrGposteriororbitalgyrus	0.1061	0.1208	0.0012	-0.6449
RightFRPfrontalpole	0.0852	0.0989	0.0306	-0.3944
RightPrGprecentralgyrus	0.2414	0.2689	0.0001	-0.7743
RightMPrGprecentralgyrusmedialsegment	0.1860	0.2025	0.0081	-0.5016
RightCOcentraloperculum	0.1514	0.1658	0.0205	-0.4331
RightSCAsubcallosalarea	0.0552	0.0646	0.0010	-0.6047
RightSFGsuperiorfrontalgyrus	0.2487	0.2706	0.0046	-0.5377
RightAOrGanteriororbitalgyrus	0.1163	0.1191	0.6430	-0.0856
RightSMCsupplementarymotorcortex	0.1847	0.1871	0.7219	-0.0669
RightFOfrontaloperculum	0.1137	0.1214	0.1376	-0.2704
RightMSFGsuperiorfrontalgyrusmedialsegment	0.1903	0.1971	0.2839	-0.1979
RightPPplanumpolare	0.0930	0.0904	0.5836	0.0958
RightPTplanumtemporale	0.1036	0.0986	0.3283	0.1770
RightPHGparahippocampalgyrus	0.0919	0.0942	0.6171	-0.0898
RightSTGsuperiortemporalgyrus	0.1422	0.1443	0.6422	-0.0862
RightTMPtemporalpole	0.1177	0.1189	0.7941	-0.0486
RightMTGmiddletemporalgyrus	0.1651	0.1808	0.0038	-0.5326
RightTTGtransversetemporalgyrus	0.0884	0.0811	0.0949	0.3054

RightFuGfusiformgyrus	0.1284	0.1413	0.0031	-0.5556
RightITGinferiortemporalgyrus	0.1524	0.1679	0.0043	-0.5321
RightEntentorhinalarea	0.0882	0.0922	0.2686	-0.1995
RightSPLsuperiorparietallobule	0.2002	0.2163	0.0041	-0.5448
RightSMGsupramarginalgyrus	0.1581	0.1745	0.0019	-0.5970
RightAnGangulargyrus	0.1738	0.1898	0.0017	-0.5734
RightPCuprecuneus	0.1985	0.2084	0.0901	-0.3114
RightPOparietaloperculum	0.1195	0.1229	0.5023	-0.1241
RightPoGpostcentralgyrus	0.2007	0.2205	0.0023	-0.5909
RightMPoGpostcentralgyrusmedialsegment	0.1083	0.1375	0.0000	-0.9850
RightSOGsuperioroccipitalgyrus	0.1670	0.1708	0.4489	-0.1377
RightCalccalcarinecortex	0.1648	0.1612	0.5167	0.1168
RightCuncuneus	0.1638	0.1599	0.4650	0.1291
RightOCPoccipitalpole	0.1215	0.1200	0.7119	0.0670
RightLiGlingualgyrus	0.1482	0.1481	0.9742	0.0058
RightOFuGoccipitalfusiformgyrus	0.1412	0.1440	0.5216	-0.1166
RightIOGinferioroccipitalgyrus	0.1536	0.1569	0.4423	-0.1386
RightMOGmiddleoccipitalgyrus	0.1380	0.1467	0.0403	-0.3748
RightPCgGposteriorcingulategyrus	0.1845	0.1912	0.2171	-0.2242
RightMCgGmiddlecingulategyrus	0.1689	0.1715	0.7003	-0.0681
RightACgGanteriorcingulategyrus	0.1499	0.1523	0.6354	-0.0883
RightPInsposteriorinsula	0.1267	0.1216	0.2205	0.2165
RightAInsanteriorinsula	0.1424	0.1511	0.0919	-0.3124
RightCerebellumExterior	0.2247	0.2542	0.0000	-0.9553
Pons	0.0232	0.0326	0.0062	-0.5850
BrainStem	0.1733	0.1876	0.0036	-0.5462
CerebellarVermalLobulesI-V	0.1867	0.2083	0.0000	-0.7953
CerebellarVermalLobulesVI-VII	0.1205	0.1235	0.4661	-0.1259
CerebellarVermalLobulesVIII-X	0.1284	0.1384	0.0256	-0.4171

Table 2: Results of the statistical and effect size analysis of the *weighted nodal efficiency* E_i of the structural brain networks of EP and FT.

6

Region	Mean EP	Mean FT	p-value	Cohen's ds
LeftAccumbensArea	0.0002	0.0000	0.3203	0.1406
LeftThalamusProper	0.1378	0.1408	0.7223	-0.0602
LeftPutamen	0.0630	0.0811	0.0156	-0.4466
LeftPallidum	0.0000	0.0000	1.0000	0.0000
LeftBasalForebrain	0.0000	0.0000	1.0000	0.0000
LeftCaudate	0.0323	0.0418	0.1546	-0.2671
LeftAmygdala	0.0000	0.0000	1.0000	0.0000
LeftHippocampus	0.0415	0.0530	0.0431	-0.3776
LeftMOrGmedialorbitalgyrus	0.0243	0.0200	0.1203	0.2779

LeftGRegyrusrectus	0.0018	0.0026	0.4531	-0.1419
LeftTrIFGtriangularpartoftheinferiorfrontalgyrus	0.0098	0.0081	0.4406	0.1425
LeftMFGmiddlefrontalgyrus	0.1623	0.1363	0.1021	0.2963
LeftMFCmedialfrontalcortex	0.0186	0.0138	0.1092	0.2670
LeftOpIFGopercularpartoftheinferiorfrontalgyrus	0.0089	0.0105	0.3336	-0.1735
LeftLOrGlateralorbitalgyrus	0.0027	0.0020	0.4575	0.1287
LeftOrIFGorbitalpartoftheinferiorfrontalgyrus	0.0004	0.0001	0.2053	0.1807
LeftPOrGposteriororbitalgyrus	0.0050	0.0067	0.2789	-0.1955
LeftFRPfrontalpole	0.0000	0.0000	1.0000	0.0000
LeftPrGprecentralgyrus	0.1941	0.2397	0.0009	-0.6322
LeftMPrGprecentralgyrusmedialsegment	0.0049	0.0029	0.1283	0.2462
LeftCOcentraloperculum	0.0059	0.0040	0.3041	0.1557
LeftSCAsubcallosalarea	0.0000	0.0000	1.0000	0.0000
LeftSFGsuperiorfrontalgyrus	0.2133	0.2092	0.8297	0.0374
LeftAOrGanteriororbitalgyrus	0.0029	0.0009	0.0965	0.2641
LeftSMCsupplementarymotorcortex	0.0071	0.0027	0.1126	0.2576
LeftFOfrontaloperculum	0.0005	0.0001	0.1682	0.1997
LeftMSFGsuperiorfrontalgyrusmedialsegment	0.0280	0.0181	0.0129	0.4125
LeftPPplanumpolare	0.0029	0.0033	0.7620	-0.0494
LeftPTplanumtemporale	0.0012	0.0024	0.2415	-0.2240
LeftPHGparahippocampalgyrus	0.0005	0.0012	0.1981	-0.2638
LeftSTGsuperiortemporalgyrus	0.0419	0.0401	0.4921	0.1287
LeftTMPtemporalpole	0.0181	0.0154	0.3042	0.1857
LeftMTGmiddletemporalgyrus	0.0722	0.0718	0.9472	0.0122
LeftTTGtransversetemporalgyrus	0.0000	0.0001	0.3220	-0.2261
LeftFuGfusiformgyrus	0.0099	0.0099	0.9772	0.0051
LeftITGinferiortemporalgyrus	0.0274	0.0270	0.8940	0.0244
LeftEntentorhinalarea	0.0030	0.0025	0.5689	0.1012
LeftSPLsuperiorparietallobule	0.1451	0.1407	0.6399	0.0817
LeftSMGsupramarginalgyrus	0.0396	0.0290	0.0581	0.3300
LeftAnGangulargyrus	0.0809	0.0749	0.4229	0.1415
LeftPCuprecuneus	0.0995	0.0868	0.1813	0.2340
LeftPOparietaloperculum	0.0024	0.0008	0.2306	0.1846
LeftPoGpostcentralgyrus	0.0832	0.0590	0.0169	0.4232
LeftMPoGpostcentralgyrusmedialsegment	0.0000	0.0000	1.0000	0.0000
LeftSOGsuperioroccipitalgyrus	0.0421	0.0395	0.5678	0.1019
LeftCalccalcarinecortex	0.0196	0.0157	0.0482	0.3454
LeftCuncuneus	0.0193	0.0165	0.3667	0.1498
LeftOCPoccipitalpole	0.0002	0.0002	0.7655	0.0500
LeftLiGlingualgyrus	0.0064	0.0036	0.0034	0.4763
LeftOFuGoccipitalfusiformgyrus	0.0064	0.0053	0.3442	0.1703
LeftIOGinferioroccipitalgyrus	0.0165	0.0187	0.3388	-0.1638
LeftMOGmiddleoccipitalgyrus	0.0265	0.0263	0.9636	0.0079
LeftPCgGposteriorcingulategyrus	0.0339	0.0195	0.0097	0.3974
LeftMCgGmiddlecingulategyrus	0.0228	0.0232	0.9672	-0.0085
LeftACgGanteriorcingulategyrus	0.0236	0.0217	0.6140	0.0845
LeftPInsposteriorinsula	0.0000	0.0003	0.1716	-0.3135
LeftAInsanteriorinsula	0.0066	0.0049	0.3748	0.1534

LeftCerebellumExterior	0.0787	0.0664	0.1258	0.2377
RightAccumbensArea	0.0001	0.0003	0.4704	-0.1581
RightThalamusProper	0.1409	0.1435	0.7585	-0.0494
RightPutamen	0.0690	0.0800	0.1042	-0.3084
RightPallidum	0.0002	0.0000	0.3203	0.1406
RightBasalForebrain	0.0000	0.0004	0.2256	-0.2773
RightCaudate	0.0192	0.0350	0.0014	-0.6507
RightAmygdala	0.0002	0.0002	0.9430	0.0118
RightHippocampus	0.0267	0.0361	0.0921	-0.3118
RightMOrGmedialorbitalgyrus	0.0374	0.0285	0.0309	0.4025
RightGRegyrusrectus	0.0031	0.0058	0.2149	-0.2553
RightTrIFGtriangularpartoftheinferiorfrontalgyrus	0.0047	0.0056	0.5491	-0.1068
RightMFGmiddlefrontalgyrus	0.1496	0.1131	0.0088	0.4488
RightMFCmedialfrontalcortex	0.0113	0.0109	0.8839	0.0245
RightOpIFGopercularpartoftheinferiorfrontalgyrus	0.0102	0.0096	0.6645	0.0761
RightLOrGlateralorbitalgyrus	0.0032	0.0032	0.9556	0.0097
RightOrIFGorbitalpartoftheinferiorfrontalgyrus	0.0005	0.0001	0.1362	0.2120
RightPOrGposteriororbitalgyrus	0.0053	0.0077	0.1873	-0.2418
RightFRPfrontalpole	0.0000	0.0003	0.3220	-0.2261
RightPrGprecentralgyrus	0.1889	0.2314	0.0057	-0.5429
RightMPrGprecentralgyrusmedialsegment	0.0061	0.0013	0.0003	0.5496
RightCOcentraloperculum	0.0023	0.0028	0.7051	-0.0750
RightSCAsubcallosalarea	0.0000	0.0000	0.1593	-0.3230
RightSFGsuperiorfrontalgyrus	0.2645	0.2450	0.2894	0.1903
RightAOrGanteriororbitalgyrus	0.0021	0.0023	0.9378	-0.0145
RightSMCsupplementarymotorcortex	0.0046	0.0023	0.1582	0.2321
RightFOfrontaloperculum	0.0002	0.0000	0.3760	0.1255
RightMSFGsuperiorfrontalgyrusmedialsegment	0.0282	0.0265	0.7493	0.0551
RightPPplanumpolare	0.0009	0.0003	0.4739	0.1036
RightPTplanumtemporale	0.0000	0.0007	0.1524	-0.3285
RightPHGparahippocampalgyrus	0.0000	0.0000	0.1802	0.1923
RightSTGsuperiortemporalgyrus	0.0473	0.0463	0.4256	0.1427
RightTMPtemporalpole	0.0276	0.0195	0.0006	0.6278
RightMTGmiddletemporalgyrus	0.0419	0.0452	0.5284	-0.1091
RightTTGtransversetemporalgyrus	0.0000	0.0000	0.3203	0.1406
RightFuGfusiformgyrus	0.0068	0.0086	0.4678	-0.1193
RightITGinferiortemporalgyrus	0.0241	0.0315	0.0393	-0.3868
RightEntentorhinalarea	0.0026	0.0022	0.7475	0.0577
RightSPLsuperiorparietallobule	0.1494	0.1416	0.4537	0.1283
RightSMGsupramarginalgyrus	0.0194	0.0173	0.6723	0.0796
RightAnGangulargyrus	0.0641	0.0517	0.0639	0.3234
RightPCuprecuneus	0.1254	0.1214	0.6716	0.0715
RightPOparietaloperculum	0.0007	0.0012	0.6503	-0.0795
RightPoGpostcentralgyrus	0.0604	0.0518	0.4537	0.1360
RightMPoGpostcentralgyrusmedialsegment	0.0000	0.0000	1.0000	0.0000
RightSOGsuperioroccipitalgyrus	0.0519	0.0477	0.4482	0.1327
RightCalccalcarinecortex	0.0242	0.0199	0.0584	0.3471
RightCuncuneus	0.0303	0.0240	0.0582	0.3327

RightOCPoccipitalpole	0.0001	0.0000	0.2005	0.1952
RightLiGlingualgyrus	0.0059	0.0050	0.4513	0.1269
RightOFuGoccipitalfusiformgyrus	0.0073	0.0057	0.3352	0.1592
RightIOGinferioroccipitalgyrus	0.0200	0.0208	0.7521	-0.0560
RightMOGmiddleoccipitalgyrus	0.0063	0.0057	0.7682	0.0494
RightPCgGposteriorcingulategyrus	0.0322	0.0215	0.0488	0.3405
RightMCgGmiddlecingulategyrus	0.0240	0.0196	0.6321	0.0976
RightACgGanteriorcingulategyrus	0.0297	0.0208	0.0872	0.2727
RightPInsposteriorinsula	0.0000	0.0001	0.5556	-0.1228
RightAInsanteriorinsula	0.0074	0.0044	0.1290	0.2425
RightCerebellumExterior	0.0753	0.0681	0.3874	0.1347
Pons	0.0000	0.0000	1.0000	0.0000
BrainStem	0.0048	0.0005	0.1839	0.1893
CerebellarVermalLobulesI-V	0.0090	0.0107	0.2878	-0.1938
CerebellarVermalLobulesVI-VII	0.0000	0.0000	1.0000	0.0000
CerebellarVermalLobulesVIII-X	0.0000	0.0000	1.0000	0.0000

Table 3: Results of the statistical and effect size analysis of the *weighted betweenness centrality* B_i of the structural brain networks of EP and FT.

7

2 Distribution of brain volume

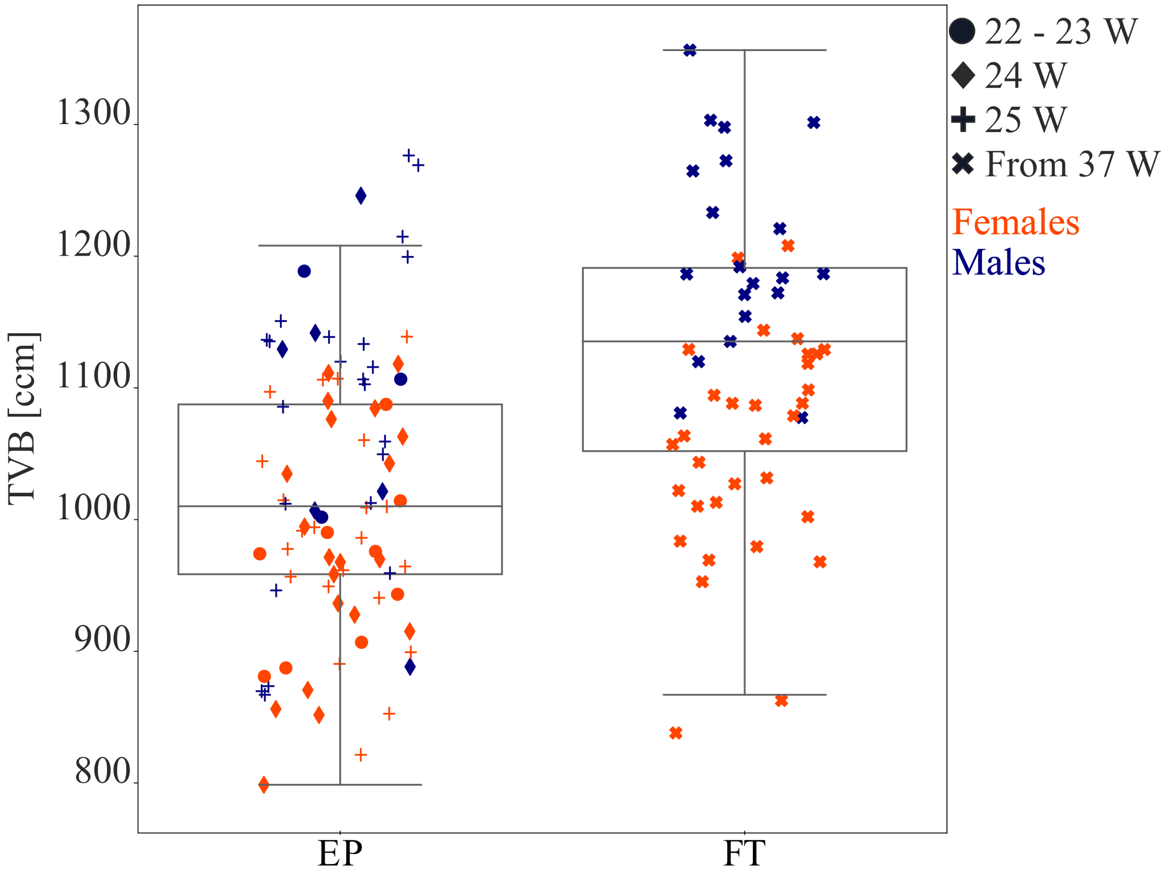

Figure 1: The distribution of the Total Brain Volume (TBV) for Extremely preterm (EP) and Full-Term (FT) born subjects. The samples are displayed with different colours to differentiate by sex and characters to separate between gestational age in completed Weeks (W). The FT group has higher TBV compared to EP group.

3 Linear regression: impact of adding the volume of ROIs as a regressor

In the main manuscript, we examine the effect of TBV and prematurity on each graph measure by using a linear model in which, for each brain region, the graph theory measure is the dependent variable, and TBV and prematurity are the regressors. For the rest of the discussion, we refer to this model as model 1. The main result of model 1 is that the brain volume explains a large portion of the effects of the network measures. We investigate if the remaining effects in these measures, which in model 1 are explained by group membership, can be explained by the volume of the specific brain regions (ROIs). We build a second linear model with an additional univariate regressor, namely the specific volume of ROI. We refer to this model as model 2. This model is repeatedly applied for each

ROI, keeping TBV and prematurity regressors unchanged. The results for statistical significance and variance explained by each regressor in model 2 are shown in Figures 2 and 3. The comparison between Figures 2 and 3 below (depicting results of model 2) and Figures 5 and 6 in the main manuscript (depicting results of model 1) overall shows that in model 2, the effect of TBV subdivides into TBV and single ROIs, as expected, leaving the effect of prematurity almost unchanged. In model 2, the connectivity strength still shows highly significant differences in the deep nuclei, with a limited decrease in significance only for the caudate. A limited decrease in the significance of differences in the deep nuclei is also observed for the nodal efficiency, which nevertheless maintains the trends. Contrarily, betweenness centrality shows a shift of significance from the effect of TBV to prematurity in model 2. The maps of the variance explained are consistent with and support these findings, as expected.

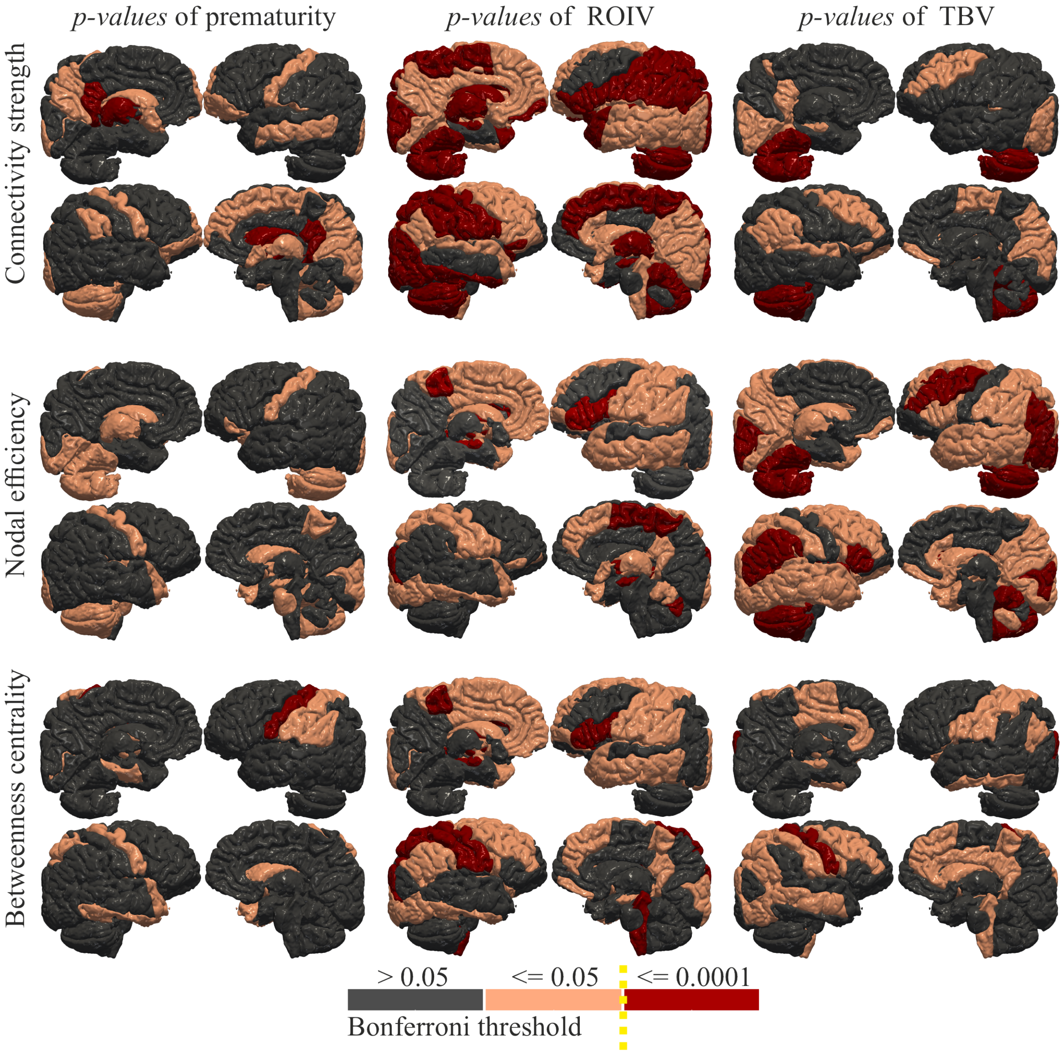

Figure 2: Significance map of the effect of extreme preterm birth (prematurity), volume of individual brain regions (ROIV), and Total Brain Volume (TBV) on each graph metric. The colour scale for statistical significance ranges from grey when the node is not statistically significance, light red when the node is significant at $p-$value lower than 0.05, to dark red when the node is significant after Bonferroni correction.

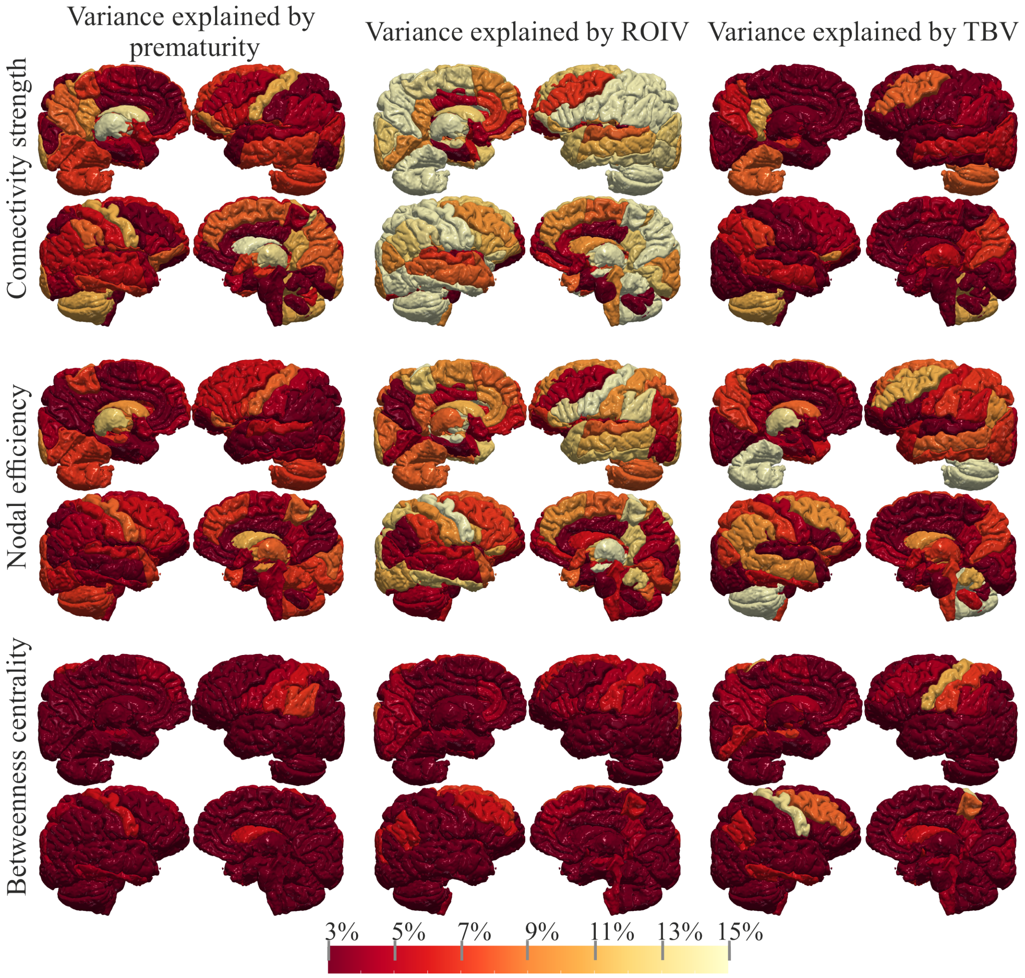

Figure 3: Maps of variance explained by extreme preterm birth (prematurity), volume of individual brain regions (ROIV), and Total Brain Volume (TBV) on each graph metric. The colour bar shows that the brighter the colour, the higher the variance explained.

The linear model 2, containing the volume of ROI as a regressor, might suffer from collinearity issues. This problem occurs when two or more regressors are correlated as the TBV and the ROI volume are. Although multicollinearity might not be a concern for the model's power of prediction, it inflates the variance of the affected variables, namely, TBV and ROIs volume. As bigger TBV directs to a bigger volume of ROI, these two variables move in the same direction. This pattern makes it hard for the model to tease apart the single effect, which drives the standard error high. To quantitatively analyse the potential multicollinearity, we investigate the correlation between TBV and the volume of ROIs, and estimate how the TBV regressor is affected by adding the ROI volume as a regressor. Figure 4.A below shows that the correlation between TBV and ROIs' volume is undoubtedly high (above 0.5 for 90% of the regions). Although there is no clear rule when a correlation is too high to lead to multicollinearity, these values indicate that there is risk of multicollinearity. To investigate how adding ROI's volume as a regressor affects TBV regressor, we compare the ratio between the

coefficient and standard error of TBV in model 1 and model 2, as the standard error would be inflated in the case of multicollinearity. We compare the ratio of the coefficient of TBV and its standard error in model 1 and model 2. The results are shown in Figure 4.B below. The figure suggests that the standard error of TBV in model 2 increases with respect to model 1. This result might hint that, in model 2, it becomes harder to disentangle the effect of ROI's volume from TBV. For the reasons listed above, we think that model 2 does not constitute an improvement for describing the relationship between prematurity, TBV, and graph measures.

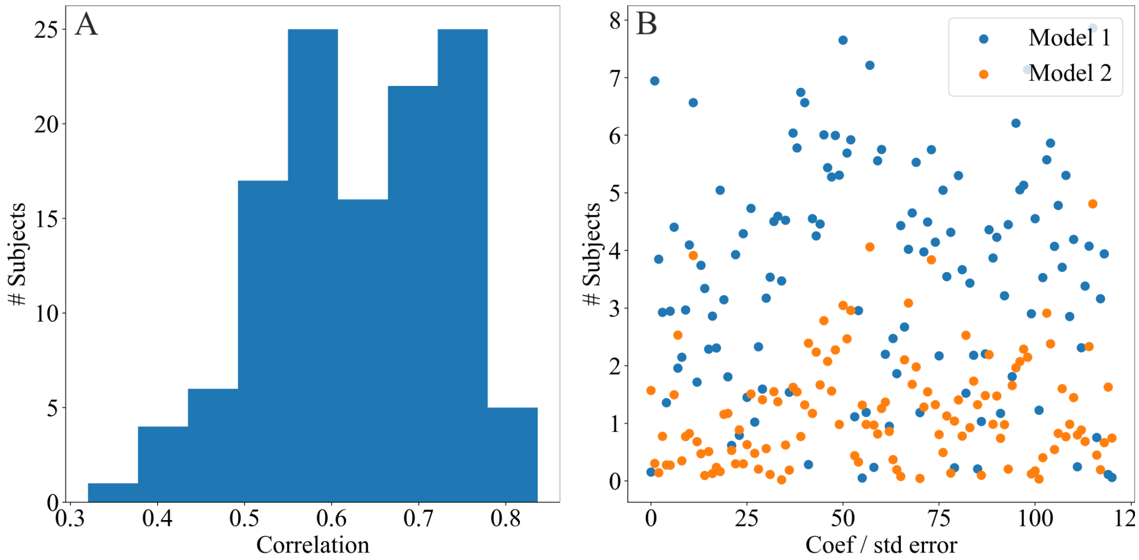

Figure 4: A: Distribution of the correlation values between the Total Brain Volume (TBV) and volume of individual ROIs. B: Scatter plot of the analysis of the ratio of the coefficient and standard error of TBV in model 1 and 2. The ratio is computed for each volume of ROI.

4 Hub regions

Hubs in FT	Hubs in EP
LeftThalamusProper	LeftThalamusProper
LeftPutamen	LeftPutamen
LeftCaudate	LeftCaudate
LeftMFGmiddlefrontalgyrus	LeftMFGmiddlefrontalgyrus
LeftPrGprecentralgyrus	LeftPrGprecentralgyrus
LeftSFGsuperiorfrontalgyrus	LeftSFGsuperiorfrontalgyrus
LeftSMCsupplementarymotorcortex	LeftSMCsupplementarymotorcortex
-	LeftMSFGsuperiorfrontalgyrusmedialsegment
LeftMTGmiddletemporalgyrus	LeftMTGmiddletemporalgyrus
LeftSPLsuperiorparietallobule	LeftSPLsuperiorparietallobule
LeftAnGangulargyrus	LeftAnGangulargyrus
LeftPCuprecuneus	LeftPCuprecuneus
LeftPoGpostcentralgyrus	LeftPoGpostcentralgyrus
LeftCerebellumExterior	LeftCerebellumExterior
RightThalamusProper	RightThalamusProper
RightPutamen	RightPutamen
RightCaudate	RightCaudate
RightMFGmiddlefrontalgyrus	RightMFGmiddlefrontalgyrus
RightPrGprecentralgyrus	RightPrGprecentralgyrus
RightSFGsuperiorfrontalgyrus	RightSFGsuperiorfrontalgyrus
-	RightSMCsupplementarymotorcortex
RightMTGmiddletemporalgyrus	RightMTGmiddletemporalgyrus
RightITGinferiortemporalgyrus	-
RightSPLsuperiorparietallobule	RightSPLsuperiorparietallobule
RightAnGangulargyrus	RightAnGangulargyrus
RightPCuprecuneus	RightPCuprecuneus
RightPoGpostcentralgyrus	RightPoGpostcentralgyrus
RightPCgGposteriorcingulategyrus	RightPCgGposteriorcingulategyrus
RightCerebellumExterior	RightCerebellumExterior

Table 4: List of the identified putative hubs for Full-Term (FT) and Extremely Preterm (EP) subjects.

5 Peripheral regions

Peripheral regions in FT	Peripheral regions in EP
LeftAccumbensArea	LeftAccumbensArea
LeftBasalForebrain	LeftBasalForebrain
LeftAmygdala	LeftAmygdala
LeftLOrGlateralorbitalgyrus	LeftLOrGlateralorbitalgyrus
LeftOrIFGorbitalpartoftheinferiorfrontalgyrus	LeftOrIFGorbitalpartoftheinferiorfrontalgyrus
LeftFRPfrontalpole	LeftFRPfrontalpole
LeftSCAsubcallosalarea	LeftSCAsubcallosalarea
LeftAOrGanteriororbitalgyrus	-
LeftTTGtransversetemporalgyrus	LeftTTGtransversetemporalgyrus
LeftEntentorhinalarea	LeftEntentorhinalarea
LeftMPoGpostcentralgyrusmedialsegment	LeftMPoGpostcentralgyrusmedialsegment
LeftOCPoccipitalpole	-
LeftPInsposteriorinsula	LeftPInsposteriorinsula
RightAccumbensArea	RightAccumbensArea
RightPallidum	RightPallidum
RightBasalForebrain	RightBasalForebrain
RightAmygdala	RightAmygdala
-	RightGRegyrusrectus
RightLOrGlateralorbitalgyrus	RightLOrGlateralorbitalgyrus
RightOrIFGorbitalpartoftheinferiorfrontalgyrus	RightOrIFGorbitalpartoftheinferiorfrontalgyrus
RightFRPfrontalpole	RightFRPfrontalpole
RightSCAsubcallosalarea	RightSCAsubcallosalarea
RightAOrGanteriororbitalgyrus	-
RightPPplanumpolare	RightPPplanumpolare
RightPTplanumtemporale	RightPTplanumtemporale
RightPHGparahippocampalgyrus	RightPHGparahippocampalgyrus
RightTTGtransversetemporalgyrus	RightTTGtransversetemporalgyrus
RightEntentorhinalarea	RightEntentorhinalarea
RightMPoGpostcentralgyrusmedialsegment	RightMPoGpostcentralgyrusmedialsegment
Pons	Pons
CerebellarVermalLobulesVI-VII	CerebellarVermalLobulesVI-VII

Table 5: List of the identified peripheral regions for Full-Term (FT) born and Extremely Preterm (EP) subjects.

6 Microstructural features

	Prematurity		TBV	
	p-value	Var. explained	p-value	Var. explained
FA				
Hubs	$1.14e^{-1}$	3.8%	$2.58e^{-7}$	21.7%
Peripheral regions	$5.77e^{-2}$	4.9%	$1.76e^{-7}$	22.3%
Global	$4.13e^{-2}$	5.4%	$1.93e^{-7}$	22.2%
MD $*1e^{-3}$[mm^2/s]				
Hubs	$2.38e^{-4}$	12.1%	$2.86e^{-1}$	2.0%
Peripheral regions	$3.18e^{-4}$	11.4%	$4.63e^{-1}$	1.2%
Global	$6.46e^{-4}$	10.7%	$2.18e^{-1}$	2.4%
NDI				
Hubs	$6.56e^{-2}$	3.0%	$9.22e^{-1}$	0.1%
Peripheral regions	$4.03e^{-2}$	3.8%	$8.24e^{-1}$	0.2%
Global	$5.98e^{-2}$	3.3%	$7.92e^{-1}$	0.2%
ODI				
Hubs	$2.61e^{-2}$	1.3%	$3.95e^{-17}$	44.3%
Peripheral regions	$8.51e^{-1}$	0.3%	$1.07e^{-13}$	38.1%
Global	$4.07e^{-1}$	1.0%	$2.23e^{-16}$	43.9%

Table 6: Results of covariate analysis for evaluating the effect of being born extremely preterm (Prematurity) and Total Brain Volume (TBV) on the values of Fractional Anisotropy (FA), Mean Diffusivity (MD), Neurite Density Index (NDI), and Orientation Dispersion Index (ODI) over the brain and along hub and peripheral sub-networks.

Parameters	Statistics	Completed gestational weeks		
**Hubs**		23 vs 24	23 vs 25	24 vs 25
**FA**	median (IQR)	0.447 (0.040) vs 0.454 (0.028)	0.447 (0.040) vs 0.466 (0.029)	0.454 (0.028) vs 0.466 (0.029)
	95% CI	(-0.019, 0.021)	(-0.028, 0.007)	(-0.025, -0.002)
	p-value	0.671	0.158	**0.021**
**MD** e^{-3} mm^2/s	median (IQR)	0.708 (0.069) vs 0.692 (0.043)	0.708 (0.069) vs 0.682 (0.037)	0.692 (0.043) vs 0.682 (0.037)
	95% CI	(-0.028, 0.038)	(-0.01, 0.047)	(-0.005, 0.028)
	p-value	0.777	0.302	0.160
**ODI**	median (IQR)	0.262 (0.016) vs 0.262 (0.015)	0.262 (0.016) vs 0.256 (0.011)	0.262 (0.015) vs 0.256 (0.011)
	95 % CI	(-0.011, 0.005)	(-0.003, 0.009)	(0.001, 0.011)
	p-value	0.451	0.282	**0.014**
**NDI**	median (IQR)	0.575 (0.049) vs 0.590 (0.039)	0.575 (0.049) vs 0.586 (0.039)	0.590 (0.039) vs 0.586 (0.039)
	95% CI	(-0.032, 0.015)	(-0.036, 0.009)	(-0.019, 0.015)
	p-value	0.370	0.273	0.594
**Peripheral regions**				
**FA**	median (IQR)	0.410 (0.040) vs 0.408 (0.031)	0.410 (0.040) vs 0.424 (0.036)	0.408 (0.031) vs 0.424 (0.036)
	95% CI	(-0.016, 0.023)	(-0.026, 0.009)	(-0.025, -0.003)
	p-value	0.741	0.221	**0.014**
**MD** e^{-3} mm^2/s	median (IQR)	0.709 (0.043) vs 0.692 (0.050)	0.709 (0.043) vs 0.684 (0.039)	0.692 (0.050) vs 0.684 (0.039)
	95% CI	(-0.015, 0.034)	(-0.008, 0.034)	(-0.013, 0.018)
	p-value	0.480	0.229	0.706
**ODI**	median (IQR)	0.288 (0.026) vs 0.287 (0.017)	0.288 (0.026) vs 0.281 (0.015)	0.287 (0.017) vs 0.281 (0.015)
	95 % CI	(-0.015, 0.006)	(-0.004, 0.014)	(0.003, 0.015)
	p-value	0.37	0.246	**0.003**
**NDI**	median (IQR)	0.561 (0.043) vs 0.566 (0.041)	0.561 (0.043) vs 0.562 (0.039)	0.566 (0.041) vs 0.562 (0.039)
	95% CI	(-0.032, 0.015)	(-0.032, 0.011)	(-0.017, 0.017)
	p-value	0.396	0.435	0.835

Table 7: The statistical comparison of microstructural parameters (FA, MD, ODI, and NDI) in hubs and peripheral sub-networks between EP sub-groups. EP are split by completed weeks of gestation as shown in table 1 of the main manuscript. The statistical tests are performed using Kruskal-Wallis test. The Interquartile Range (IQR) of the data and 95% Confidence Interval (CI) for the difference in the median are reported. The p-values in bold are bellow the typical value of 0.05. None of the values survived the Bonferroni threshold of 0.002.
